# A robust, high-content NAM for repeatable and predictive developmental and reproductive toxicity assessment in *C. elegans*

**DOI:** 10.1038/s41598-026-61532-4

**Published:** 2026-07-10

**Authors:** Sudip Mondal, Adam Laing, Amber Shen, Evan Hegarty, Abhishri Medewar, Sebastian Gomez, Gina Carrion, Julia Brown, Piper Hunt, Adela Ben-Yakar

**Affiliations:** 1 vivoVerse, LLC, Austin, TX 78731 USA; 2 U.S. Food and Drug Administration, Human Foods Program, Office of Chemistry and Toxicology, Laurel, MD 20708 USA; 3 Department of Mechanical Engineering, The University of Texas at Austin, Austin, TX 78712 USA; 4 Department of Biomedical Engineering, The University of Texas at Austin, Austin, TX 78712 USA

**Keywords:** Developmental and reproductive toxicology (DART), *C. elegans*, In utero embryo phenotype, High-resolution imaging, Microfluidic technology, Repeatable assays, Acceptance criteria, New approach methodology (NAM), Biological techniques, Biotechnology, Developmental biology

## Abstract

Developmental and reproductive toxicity (DART) assessment is essential for product safety evaluation but relies heavily on vertebrate models that are costly, time consuming, and resource intensive. Although *Caenorhabditis elegans* has emerged as a promising new approach methodology (NAM) for rapid, cost-effective, whole-organism toxicology, broader adoption has been limited by the lack of high-resolution, rapid imaging approaches, limited endpoints, and insufficient evidence supporting assay robustness. To overcome these limitations, we developed vivoDART, a multiparametric imaging-based DART assay designed to be accurate, efficient, robust, and sensitive. Building on our machine learning-accelerated developmental toxicity platform, vivoDART uses microfluidic-assisted, high-resolution brightfield imaging to quantify six developmental and reproductive endpoints for comprehensive DART analysis. Developmental endpoints are derived from automated body-dimension analysis, whereas reproductive endpoints are obtained by quantifying total in utero embryo number and classifying embryos as early- or late-stage relative to the twofold stage. The assay directly assesses sublethal DART phenotypes while minimizing reliance on non-specific apical endpoints such as lethality. vivoDART demonstrated high repeatability, with mean coefficients of variation of 1–5% for developmental endpoints and 6–17% for reproductive endpoints, supporting high statistical power. Assay validation performed using DMSO, methylmercury, and propiconazole involved phenotyping ~ 400,000 embryos across ~ 9,200 worms. Densely sampled concentration–response curves enabled EC_x_ prediction with narrow confidence intervals and captured subtle to strong effects across a broad dynamic range of 1.0–237 µM. Notably, late-stage embryo number was the most sensitive endpoint among the chemicals tested, preceding changes in total embryo number, body size, or viability. These findings indicate that the observed responses reflected DART-specific effects rather than non-specific lethality. These results establish vivoDART as a sensitive, repeatable, and scalable whole-organism platform for rapid, cost-effective chemical prioritization and comparative hazard assessment.

**Supplementary Information:**

The online version contains supplementary material available at https://doi.org/10.1038/s41598-026-61532-4.

## Introduction

Developmental and reproductive toxicity (DART) assessment is a vital part of product safety testing for chemicals, consumer products, and pharmaceuticals. Currently, most DART testing for human health risk assessment relies on mammalian models such as rats and rabbits^1,2^. However, increasing regulatory and societal pressure to reduce animal use has led to new legislative initiatives in the United States, European Union, and other regions aimed at limiting reliance on vertebrate models. In addition to ethical considerations, mammalian-based DART studies are costly, time-intensive, and resource-demanding. For example, a standard rat DART study may require 2–3 months of exposure and monitoring due to long life cycles, substantial housing requirements, and complex experimental designs. High costs also restrict sample size, potentially limiting statistical power. Despite the genetic and physiological similarities between rodents and humans, rodent data alone are only 43% predictive of human toxicity outcomes^3^. Concordance improves to 71% when rodent data are combined with data from a nonrodent species, supporting the need for multitiered testing strategies. With over 350,000 registered chemicals requiring new or updated safety evaluations^4^, there is an urgent need for New Approach Methodologies (NAMs) that can complement or, where appropriate, reduce reliance on traditional vertebrate methods.

NAM-based DART approaches include in silico prediction models^5^, in vitro systems^6–8^, ex vivo technologies^9^, and alternative invertebrate model organisms not subject to animal welfare regulations, such as *C. elegans*^10–29^, earthworms^30^, brine shrimp^31,32^, and *Daphnia*^33,34^. Although no single method can fully recapitulate human biology, a multitiered approach that integrates complementary models can provide a more comprehensive and human-relevant toxicity profile for risk assessment. In silico methods remain constrained by limited, high-quality training datasets^35^. In vitro and ex vivo systems are highly variable, often capture only isolated adverse outcome pathways (AOPs), and lack the integrated tissue- and organ-level interactions required to evaluate complex DART responses. Among invertebrate models, standardized DART assessment protocols exist for *Daphnia* and earthworms and are widely used for ecotoxicological risk assessment^34,36,37^. Although no regulatory test guidelines currently exist for *C. elegans*, this small model organism has been extensively used in scientific research, including numerous toxicology studies^11–29^.

*C. elegans* is a powerful, high-throughput model organism that can be grown quickly and inexpensively in large numbers using well-established techniques. Its small size and transparent body make it highly amenable to imaging-based methods for high-content analysis. The *C. elegans* genome and proteome shares substantial conservation with humans, with approximately 60–80% and 83% homology to the human genome and proteome, respectively^38,39^. In addition, *C. elegans* has intact and interacting neuromuscular, reproductive, and digestive systems with conserved signaling and metabolic pathways, enabling system-level AOPs studies. The reproductive system of a *C. elegans* hermaphrodite consists of a germline, somatic gonad, uterus, and egg-laying circuitry^40,41^. Conserved mechanisms governing reproduction include germ cell proliferation and differentiation, oogenesis, programmed cell death, chromosomal aneuploidy, and reproductive aging. In addition, *C. elegans* contains at least 76 cytochrome P450 genes^42^, Phase I-III xenobiotic metabolism pathways^14^, and a functional microbiome, supporting toxicological evaluation of parent compounds and metabolites with tissue-level bioavailability profiles^14,21,29^.

Previous large-scale studies have shown concordance between *C. elegans* and mammalian toxicity outcomes. Developmental toxicity endpoints in *C. elegans* demonstrate ~ 53% balanced accuracy for predicting mammalian toxicity^13^. Germline integrity studies analyzing chromosome segregation defects in *C. elegans* embryos achieved 69% balanced accuracy in predicting mammalian reproductive toxicity^23^. Acute toxicity studies of 17 non-acidic chemicals reported 88.5% concordance with rat lethal dose predictions, similar to mouse–rat concordance of 87.9%^43^. However, many of existing *C. elegans* DART-related studies rely on low-throughput, labor-intensive methods or low-resolution readouts to assess gross developmental defects and embryo outcomes following chemical exposure^44,45^, limiting scalability and reproducibility. Thus, despite evidence supporting the translational relevance of *C. elegans*, broader adoption for DART screening requires platforms that combine high-resolution phenotyping, efficiency, robustness, repeatability, and sensitivity. Recent advances in microfluidics^46–51^ and machine learning (ML)-assisted image analysis^46^ now provide an opportunity to address these limitations through robust, high-throughput, and multi-parametric analysis of *C. elegans*.

Here, we present vivoDART, a high-content imaging-based assay quantifying DART-related endpoints in *C. elegans* using a high-throughput microfluidic platform that is amenable to automation and large-scale chemical library screening^46–48^. The vivoDART assay can be completed within 3 days, from initial worm plating and chemical exposure to endpoint imaging, and all readouts are derived from brightfield image analysis. Compared with conventional DART workflows that require repeated manual transfer, multiday brood-size analysis, and separate measurements of body size, progeny number, and hatching efficiency, vivoDART enables developmental and reproductive endpoints to be quantified from intact animals within a single imaging-based workflow. The assay is compatible with wild-type strains, transgenic lines, and genetically diverse wild isolates. High-resolution 3D imaging of the intact uterus enables identification and classification of in utero embryos, providing sensitive measure of reproductive toxicity beyond body size alone. Importantly, analysis of in utero embryo development during early adulthood minimizes confounding effects associated with age-related reproductive decline and off-target effects such as altered egg-laying behavior. Using 30 replicates (5 biological × 6 technical replicates), we show that vivoDART produces highly repeatable results across multiple DART phenotypes, extended experimental periods, and independent operators. As a case study, we evaluated methylmercury and propiconazole, chemicals with well-characterized DART profiles in *Daphnia*, rodents, amphibians, and fish, to benchmark assay sensitivity and assess translational relevance.

## Results

### Unique aspects of *C. elegans*-based developmental and reproductive toxicity assay (vivoDART)

A key strength of this study is the development of vivoDART as a DART assessment platform designed to be accurate, efficient, robust, and sensitive. Among these attributes, efficiency, defined by assay throughput and processing time, is particularly important for high-content screening systems because it determines whether large chemical libraries can be evaluated at practical scale. To address this need, vivoDART combines vivoChip-based microfluidic immobilization^46–48^, high-resolution whole-organism 3D imaging, and image-based quantification of both developmental and reproductive endpoints in intact *C. elegans* following chemical exposure.

**Fig. 1:**
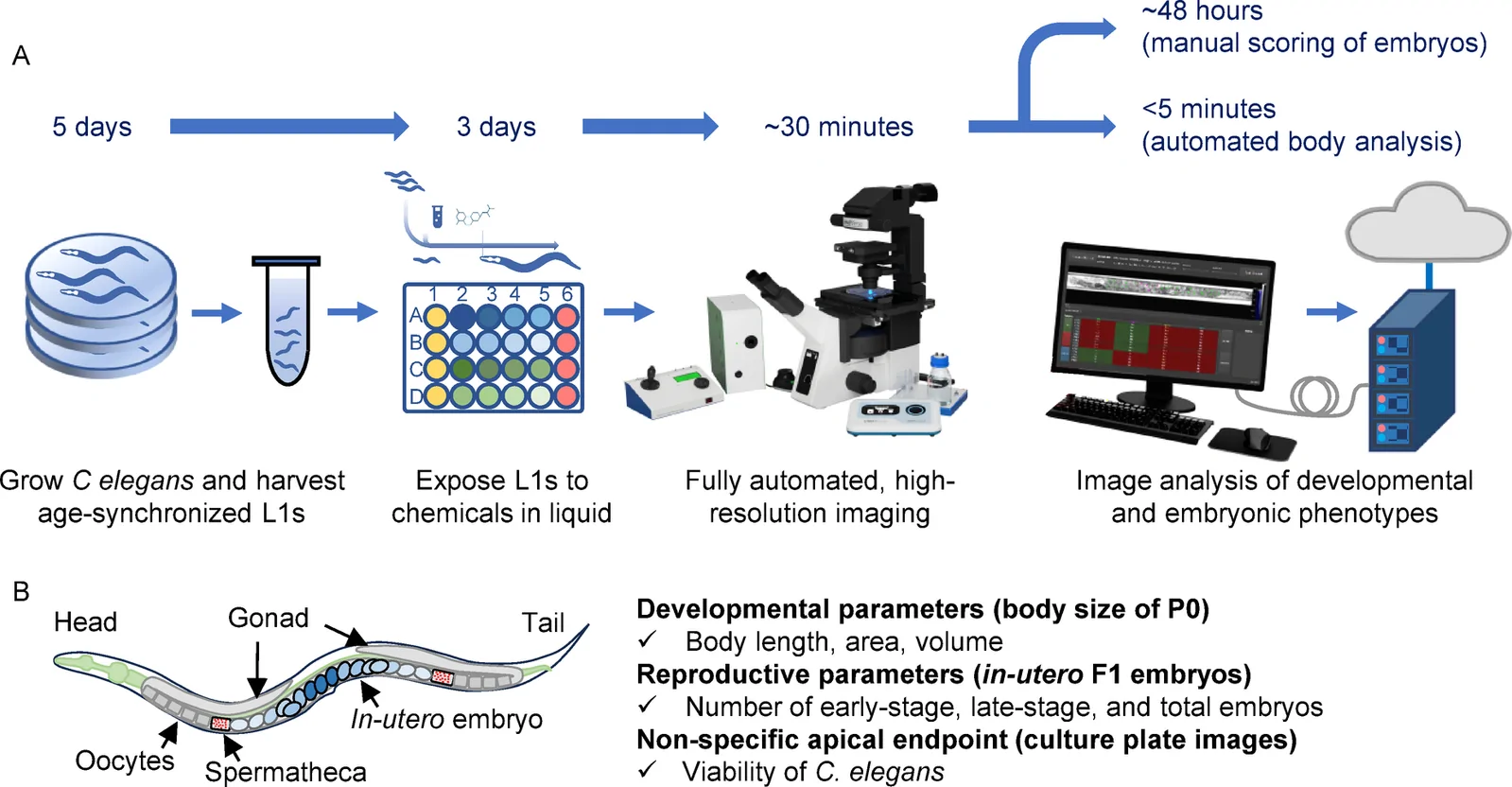
Schematic of the assay workflow for quantifying DART phenotypes in *C. elegans* following exposure to test substances. (**A**) Synchronized L1-stage *C. elegans* are cultured in liquid media containing varying concentrations of a test substance or control chemical. After 72 h of exposure, when control worms have reached the D1 adult stage, worms are loaded into the vivoChip-24x microfluidic trapping and immobilization device for rapid, high-resolution 3D imaging. Software-assisted image analysis is then used to quantify embryonic phenotypes (by manual scoring of ~ 30–40 embryos per worm across ~ 1,000 worms, requiring ~ 48 h) and developmental phenotypes (using automated ML-based quantification of body dimensions, completed in < 5 min). (**B**) Overview of DART endpoints and non-specific viability endpoint captured using vivoChip imaging data and plate images, respectively. In utero embryo number and developmental stage, indicated by different shades of blue-colored embryos, are quantified along with body development endpoints.

In the vivoDART assay, synchronized *C. elegans* populations are exposed to chemicals from the larval 1 (L1) stage for 72 h, until they reach the D1 adult stage, and are subsequently imaged using vivoChip microfluidic immobilization devices to quantify DART-related parameters (Fig. 1A). Developmental endpoints are rapidly quantified using our previously established developmental toxicity (DevTox) platform, which incorporates ML-accelerated image analysis^46^. We extended this high-content imaging and analysis approach to capture sensitive reproductive health parameters by quantifying the total number of embryos in each worm and classifying their developmental stages relative to the twofold stage. Overall, the vivoDART assay generates six DART-related endpoints: three developmental endpoints, body length, area, and volume, and three reproductive endpoints, total embryo number, early-stage embryo number, and late-stage embryo number (Fig. 1B). Total embryo number serves as an in utero proxy for the traditional brood-size metric, whereas late-stage embryo number provides an indicator of successful embryonic development and is conceptually related to hatching efficiency measurements in the traditional *C. elegans* DART assay.

The sensitivity of the vivoDART assay is enabled by whole-organism 3D imaging using the vivoChip microfluidic platform, which provides sufficient resolution to determine the developmental stage of in utero embryos inside the intact *C. elegans* uterus^52^. By directly quantifying in utero embryo number and classifying developmental progression during early adulthood*,* this assay captures sub-lethal reproductive toxicity while minimizing confounding effects associated with age-associated decline in the reproductive health and off-target effects such as altered egg-laying behavior. This approach enabled the determination of effective concentration (EC_50_) values with high confidence. In addition, brightfield imaging of *C. elegans* populations in culture plates prior to chip loading was used to confirm viability based on 10-s motility analysis of individual worms. We found that reproductive endpoints were more sensitive than developmental endpoints for the positive and reference chemicals examined in this study and, importantly, were independent of non-specific apical endpoints such as lethality.

The efficiency and robustness of vivoDART are particularly evident when compared with conventional approaches for assessing DART-related phenotypes. Conventional assays require separate measurements of body size, brood size (defined as the total number of progeny), and hatching efficiency on NGM plates^44,53^. These methods require repeated daily washing, imaging, and manual transfer of parental adults across multiple culture and imaging plates for 5–6 days, making them substantially more labor- and resource-intensive than the vivoDART assay (Supplementary Fig. 1 and Supplementary Table 1). In addition, because developmental endpoints are measured directly from parental adults whereas reproductive endpoints are inferred from laid embryos and progeny on subsequent days, conventional workflows are vulnerable to variability introduced by differences in egg-laying timing, developmental delay, and sample handling. At high chemical concentrations, severe cellular damage and fragile worm bodies can result in worm death during manual transfer. Furthermore, delayed growth and egg laying at high concentrations can prolong the experimental duration required for accurate brood size analysis. In contrast, worm populations with delayed development, small body size, and few or no laid eggs can still be reliably immobilized in the vivoChip and analyzed for in utero embryo parameters. By reducing manual handling and enabling direct analysis of developmental and reproductive endpoints, vivoDART minimizes sample loss and data heterogeneity, both of which can otherwise contribute to high variability and reduced statistical power for detecting small effect sizes in DART parameters^53^.

The primary goals of this study were to demonstrate the repeatability of the vivoDART assay, determine its statistical power to detect small effect sizes, and validate the assay using two reference chemicals. For newly developed methodologies, intra-laboratory repeatability studies represent an essential first step toward future regulatory consideration and broader validation. To test assay repeatability, experiments were performed on multiple days, using different generations of worms grown on NGM plates, and by multiple scientists. Each experiment was imaged on a vivoChip-24x device, and DART endpoints were analyzed across all 24 wells, corresponding to 24 worm populations, with ~ 40 worms per population. We used up to 40 worms in each well to calculate well-level average values for each DART parameter. Because each experiment was initiated with age-synchronized L1-stage worms derived from the same batch of worms grown on NGM plates, these isogenic worms were considered a single biological replicate. Multiple wells treated under identical conditions and imaged on the same day using the same vivoChip-24x device were considered technical replicates, whereas experiments initiated with different worm batches and imaged on different days were considered biological replicates.

We estimated assay variability using coefficient of variation (CV%) from five biological replicates and six technical replicates under four DMSO conditions. We also used 3 biological replicates for both methylmercury and propiconazole tests. In total, approximately 400,000 embryos were manually analyzed to establish the vivoDART assay and demonstrate the suitability of this approach for high-content chemical screening and sensitive detection of sub-lethal developmental and reproductive perturbations. Although manual analysis of in utero embryos was highly labor-intensive and accounted for 98% of the total assay time, requiring 72 of 74 h needed to analyze the entire dataset (Supplementary Table 1), these manually annotated datasets enabled the training of an ML model for automated embryo analysis within worm bodies. This ML-accelerated image-analysis pipeline has since been successfully implemented and validated, and the results will be reported in a separate manuscript. These advances support the development of vivoDART assay as a fully automated, cost-effective DART assessment platform using a whole-organism model.

### High-resolution, whole-body imaging for extraction of DART endpoints

**Fig. 2:**
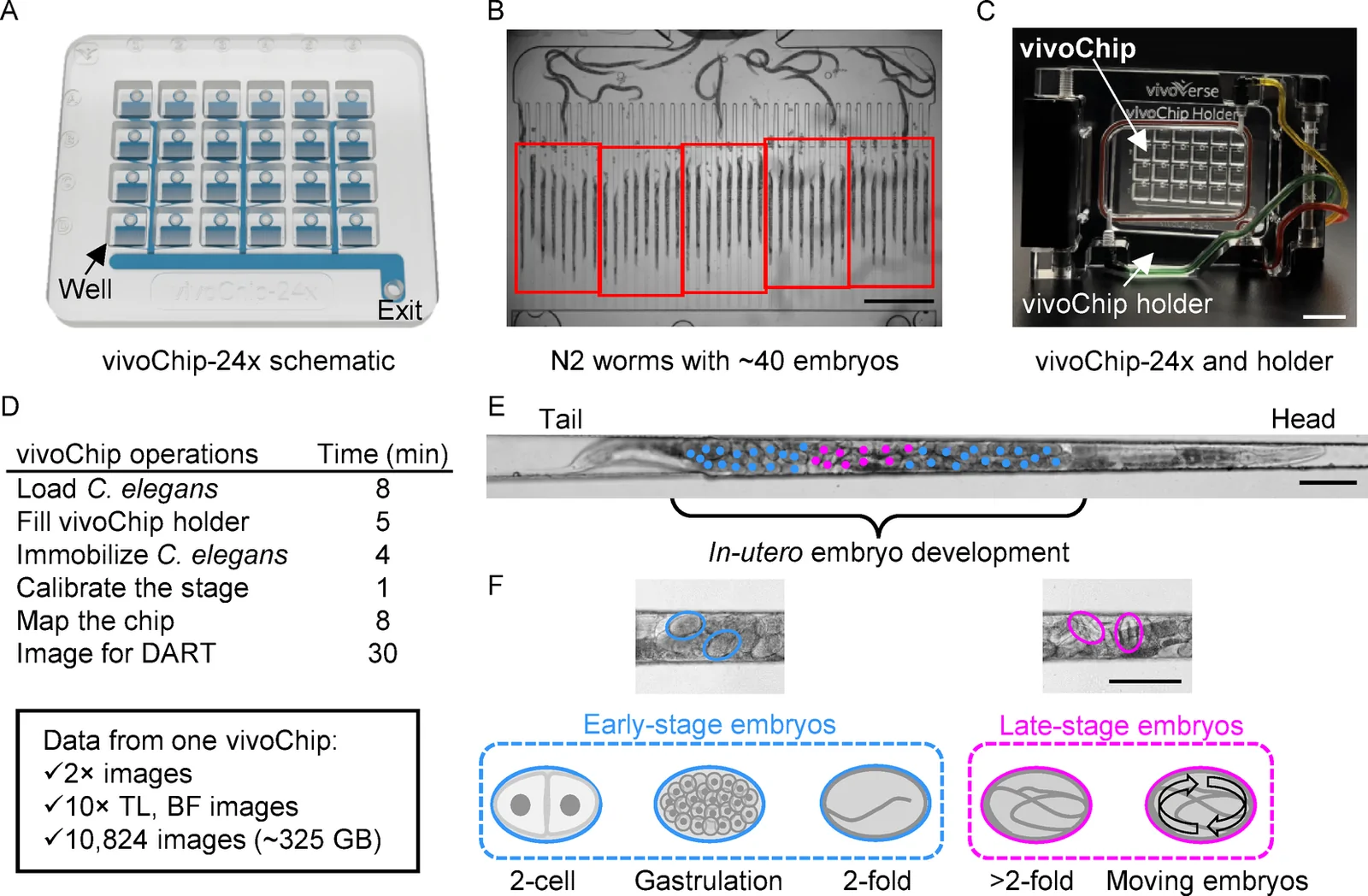
vivoChip-24x design and operation for high-resolution imaging in DART studies. (**A**) A schematic illustration of vivoChip-24x device containing 24 wells, each fluidically connected to 40 microfluidic trapping channels. (**B**) Representative image of 40 microfluidic channels under one of the 24 wells, containing N2 adult worms. Worms are trapped at different positions within the tapering channels according to body size. (**C**) The vivoChip-24x device mounted in its pressurized holder for controlled loading and immobilization. (**D**) Timeline and duration of each step in the vivoChip operation workflow and total data volume produced from a single imaging experiment. (**E**) Representative 10× image of a trapped adult worm showing 31 early-stage embryos (blue) and 10 late-stage embryos (magenta). (**F**) Representative images and schematics of embryos at developmental stages used for classification into early- and late-stage categories. Scale bars are 1 mm (**B**), 20 mm (**C**), and 100 µm (**E** and **F**).

The DART assay leverages the unique ability of the vivoChip microfluidic platform to rapidly immobilize large numbers of *C. elegans* for high-resolution whole-body imaging using the automated vivoScreen platform (see Methods, Supplementary Fig. 2). Each vivoChip-24x device enables parallel immobilization of up to 960 *C. elegans* across 24 wells, with each well containing 40 microfluidic trapping channels (Fig. 2A). The closely spaced microfluidic channels maximize information density within each field of view (FOV) (Fig. 2B). A pressurized gasket system seals the vivoChip-24x device and applies a pre-defined pressure sequence for efficient loading and immobilization (Fig. 2C). Using automated stage navigation and objective switching, all wells are imaged sequentially, and complete volumetric datasets are acquired in ~ 30 min per chip (see Methods, Fig. 2D, and Supplementary Fig. 3).

Before chip loading, movies of each well in the culture plate were captured for motility-based viability analysis. After chip loading, we performed high resolution 10× 0.4 NA brightfield z-stacks (consisting of 18 planes at 6 µm spacing) to capture the full uterus of each immobilized worm and visualize all in utero embryos across developmental stages. A single vivoChip experiment generated 10,824 images (Fig. 2D). This axial sampling strategy accommodates minor variations in worm posture within the channels while preserving imaging speed, enabling visualization of in utero embryos throughout the uterus.

Embryos were classified into two developmentally distinct categories: early-stage embryos (≤ twofold) and late-stage embryos (> twofold). The twofold stage provides a clear morphological boundary because structural complexity increases rapidly after this stage. Late-stage embryos often display fully formed larval structures, particularly in the central region of the uterus near the vulva, where embryos are frequently developed into young larvae. Visualization of larval morphology within the eggshell facilitated accurate classification. In some cases, movement within the eggshell was observable in time-lapse data (Fig. 2E–F), further supporting developmental staging. In future implementations, embryo motility could be incorporated as an additional quantitative endpoint.

### Image-analysis pipeline for robust DART phenotyping

Imaging data were first pre-processed by identifying bounding boxes around individual channels, enabling analysis of each worm-containing channel (see Methods, Supplementary Fig. 4A). Downstream image analysis consisted of two complementary workflows; (1) automated quantification of worm body dimensions using deep-learning-based segmentation (Supplementary Fig. 4B–C) and (2) semi-automated embryonic phenotyping using GUI-based analysis software to streamline manual scoring (Supplementary Fig. 4D–F).

Previously, we developed a 2.5D U-Net architecture for image-level classification and pixel-wise segmentation tasks^46^. In this architecture, both tasks were jointly learned within a single network sharing a common encoder and bottleneck, trained using three z-slices around the best-focus image. While effective, this design introduced task coupling, limiting independent modification and optimization of the two objectives. To address this limitation, we decoupled channel classification and worm segmentation into separate models, each tailored to its respective task. The classification model consisted of a convolutional encoder followed by a Vision Transformer (ViT) bottleneck and a classification head. The segmentation model consisted of a convolutional encoder, a ViT-based bottleneck, and a convolutional decoder. Since image-level classification relies primarily on global morphological features, present in the best-focus image, we trained the classification model using a single best-focus image. In contrast, pixel-wise segmentation tasks require subtle image contrast information to identify object boundaries. To improve head and tail boundary detection, we fed 5 z-stack images around the best-focus plane into the model. This approach captures features spanning multiple z-slices and improves boundary identification.

To further improve robustness under challenging imaging conditions, the training datasets for both models were expanded to include additional channels containing smaller worms located near the exit portion of the immobilization channels from both L4 and D1 vivoChip-24x devices (Supplementary Fig. 5). For classification model training, we generated ground-truth data by classifying 5,305 channels as full-worm, partial-worm, or no-worm classes. For segmentation model training, we used manually segmented masks from 4,360 channels with full worms. The classification model was evaluated on a test set of 538 images (441 full-worm, 50 partial-worm, and 47 no-worm channels) and achieved a weighted F1-score of 98.3%. The segmentation model was evaluated on a separate test set of 439 full-worm images and achieved a mean Dice score of 98.5% (Supplementary Fig. 6). High prediction accuracy for both channel classification and worm segmentation was observed for worms immobilized in D1 (Supplementary Fig. 7A–D) and L4 (Supplementary Fig. 7E–F) devices. From segmented full-worm channels, we calculated body length (defined as the longest skeleton length) and body area. Body volume was then estimated by integrating the segmented area according to the known channel height at each pixel. These three body parameters were calculated for each worm.

Embryo phenotyping is challenging because each vivoChip-24x can immobilize up to 960 adult *C. elegans,* requiring counting and classification of up to approximately 40,000 in utero embryos. Furthermore, the intertwined morphology of the uterus with the intestinal structures and gut granules requires 3D visualization of the worm uteri for accurate phenotyping. To facilitate robust embryo phenotyping, we developed an in-house, GUI-based software (vivoAnalyzer) that automatically displays each worm-containing channel in sequence, enables interactive zooming and z-stack navigation, and allows users to annotate the *xyz* centroid of each embryo while assigning early- or late-stage classification (see Methods, Supplementary Fig. 8). When necessary, the users could use time-lapse frames to confirm late-stage embryos, exhibiting larval movement. Scoring was performed by 10 trained users after validation of their scoring accuracy using a reference dataset. Worms were randomly assigned to users, and treatment conditions were blinded to minimize scoring bias. Population-level metrics for 6 DART endpoints were obtained by averaging values across worms within each well for repeatability analysis and concentration–response modeling (see methods, Supplementary Fig. 9).

### Repeatability of the DART assay

To evaluate the robustness and statistical reliability of the vivoDART assay, we studied repeatability across multiple independent experiments performed under the same test conditions. For an assay to complement or replace existing DART methods, it must be scalable, reproduceable across users and worm batches, and capable of producing repeatable results over time.

**Fig. 3:**
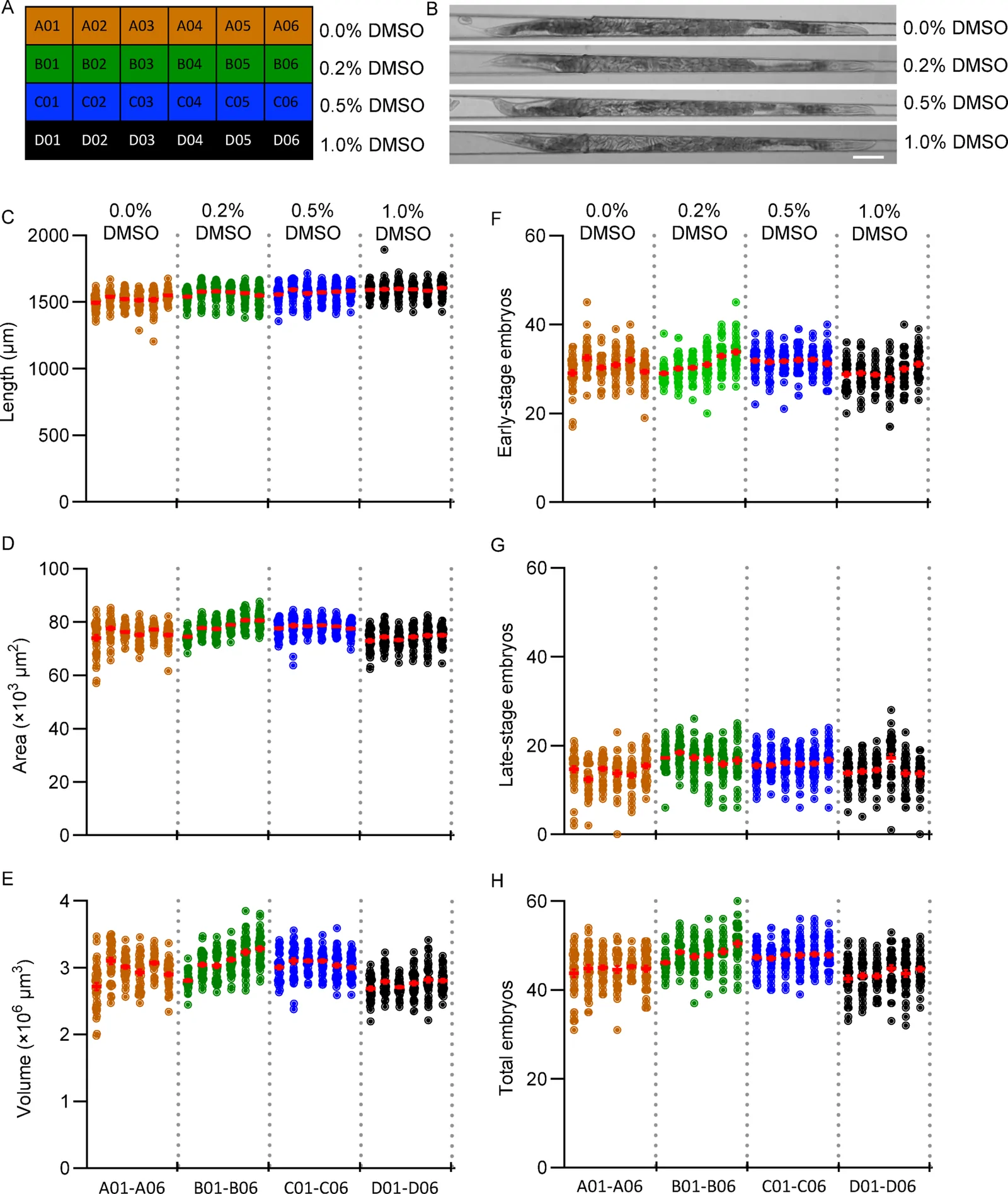
Worm-level variability in DART parameters across technical replicates from a single biological replicate. A total of 6 technical replicates were performed for each condition, corresponding to one row of the vivoChip-24x device, and 5 biological replicates were performed on different days; results from one representative biological replicate are shown. (**A**) Experimental design for a single biological replicate, containing 6 technical replicate wells for each of 4 DMSO concentrations. For each parameter, well-level averages were calculated from worm-level data obtained from up to 40 worms per well. (**B**) Representative worm images from 0%, 0.2%, 0.5%, and 1% DMSO conditions. Scale bar is 100 µm. (**C**–**H**) Scatter plots of worm-level body length (**C**), body area (**D**), body volume (**E**), number of early-stage embryos (**F**), number of late-stage embryos (**G**), and number of total embryos (**H**). Each dot represents worm-level data from an individual worm within each well after filtering for body volume and number of total embryos. The mean ± SEM values for each well are shown in red. The well-level averages (see Supplementary Fig. 10) were estimated from up to 40 worms per well. The vertical dashed lines separate the four DMSO conditions (A01-A06, B01-B06, C01-C06, and D01-D06).

We tested assay repeatability by performing 5 independent experiments (defined as biological replicates) over a two-month period using DMSO as a solvent only control. DMSO is a commonly used solvent in toxicity assays because of its wide applicability to a range of chemicals. Four DMSO concentrations (0, 0.2, 0.5, and 1.0%) were tested to characterize baseline phenotypes under solvent control conditions. Each concentration was replicated across 6 wells (defined as technical replicates) to assess well-to-well technical variability in addition to inter-experimental variability (Fig. 3A). Worms developed to the D1 adult stage across all four DMSO concentrations (Fig. 3B–E).

To examine data distribution within wells treated under a unique chemical condition, we plotted worm-level values for all 6 DART parameters from individual worms. Well-level averages for each parameter were estimated from up to 40 worms per well, all treated under the same chemical condition. We examined both worm-level distributions (Fig. 3C–H) and well-level averages (Supplementary Fig. 10) for one representative biological replicate across all 6 DART endpoints. In this biological replicate, the 6 wells treated with the same DMSO concentration, defined as technical replicates, were used to estimate aggregated average values for each DART parameter (Supplementary Fig. 10 and Supplementary Table 2). The wells with 1% DMSO produced small but statistically significant changes in body length and body volume relative to 0% DMSO, whereas body area and all embryo-related endpoints were not significantly different. Across all 5 biological replicates, well-level averages and aggregated averages calculated from all 30 wells showed similar trends for all 6 DART parameters (Supplementary Fig. 11 and Supplementary Table 3). These small changes may reflect DMSO-related effects on muscle tone, tissue elasticity, and/or interactions with the cuticle as previously reported^33^.

**Fig. 4:**
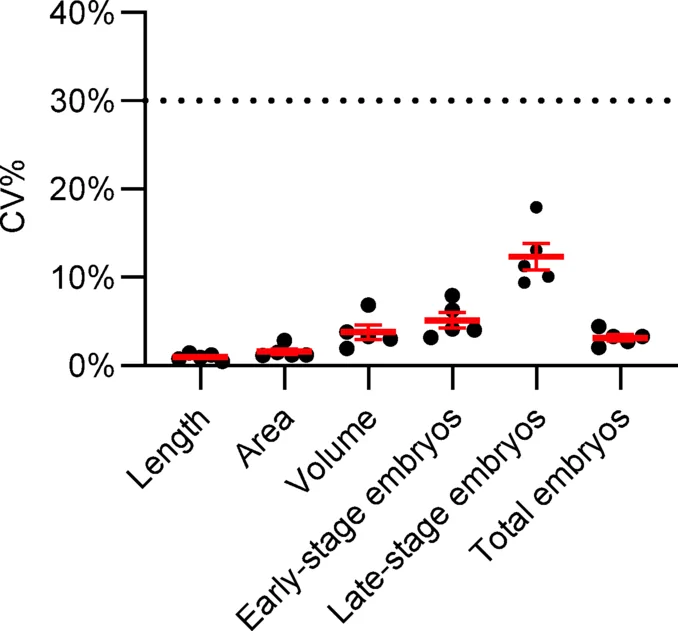
Coefficient of variation between technical replicate wells for 1% DMSO conditions. Scatter plot for the coefficient of variation (CV%) for 5 biological replicates for all 6 DART endpoints. Each data represents the CV% calculated using 6 technical replicates for 1% DMSO wells. The red line represents mean ± SEM. The dotted line represents the 30% CV line.

To evaluate technical repeatability, we calculated the CV% across the 6 technical replicate wells (columns 1 to 6 in vivoChip-24x) for each condition. For the 1% DMSO condition, mean CV% values across all 30 technical replicates were < 3.8% for 3 developmental endpoints and < 12.5% for 3 embryo-related endpoints (Fig. 4). As expected due to more mature eggs being laid first and the periodicity of egg laying, the most variable parameter was the number of late-stage embryos per adult. These low CV% values indicate high reproducibility among worm populations loaded and imaged in the vivoChip and suggest the absence of edge effects or location-based bias.

**Fig. 5:**
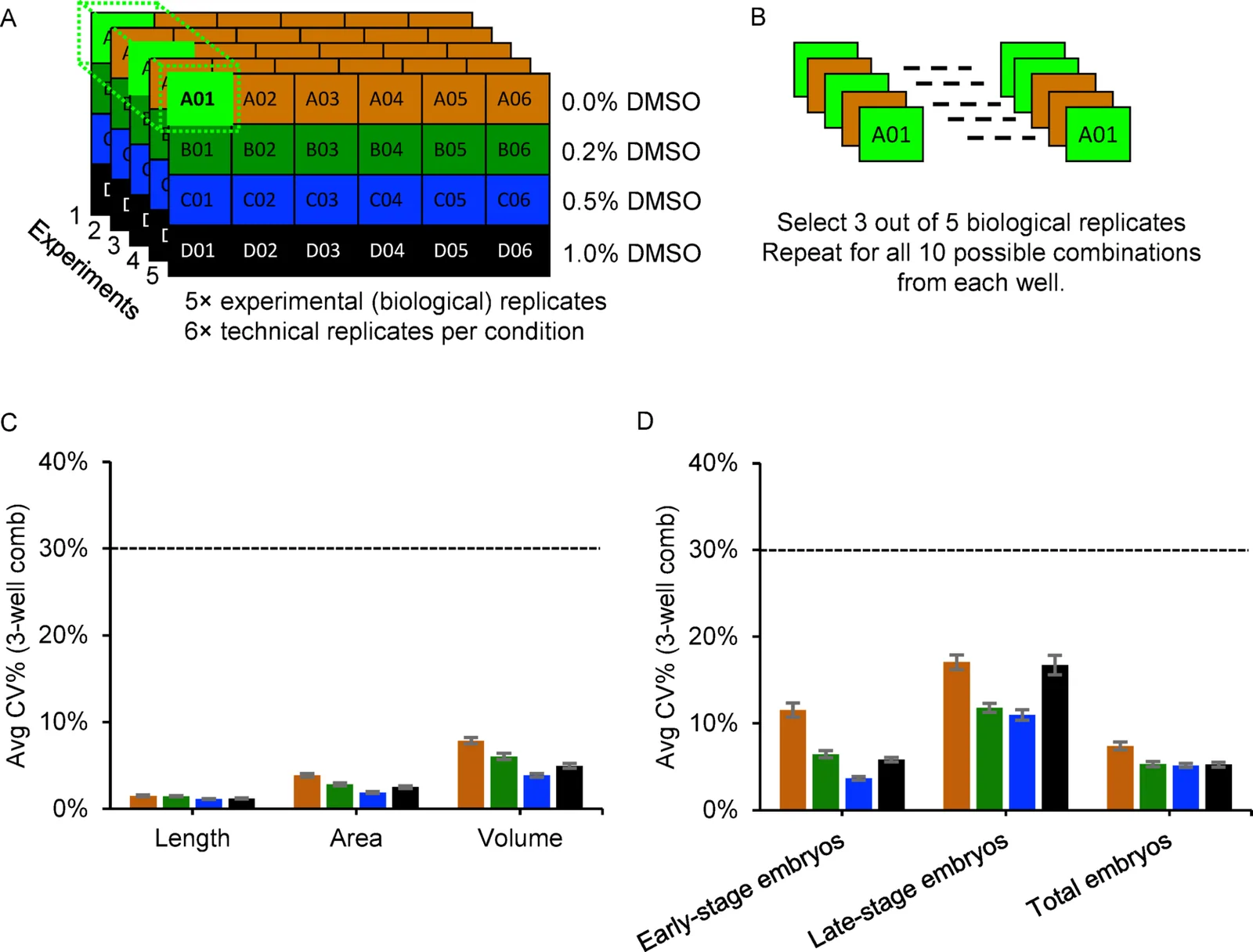
Repeatability of the DART assay across five biological replicates for all six endpoints. (**A**) Experimental design consisting of 5 independent biological replicates, each containing 6 technical replicate wells for each of 4 DMSO concentrations. (**B**) Schematic illustrating calculation of the CV% values for all possible combinations of 3 corresponding wells from 5 experiments. Each well contained up to 40 worms. (**C**) Average CV% values for body length, area, and volume from all sixty 3-well combinations. (**D**) Average CV% values for number of early-stage, late-stage, and total embryos from sixty 3-well combinations. Data presented as mean ± SEM (*n* = 60 values). The dotted line represents the 30% CV line. Individual CV% values are presented in Supplementary Fig. 12.

To assess inter-experimental repeatability, we calculated CV% values among 3 corresponding wells from 3 separate experiments (e.g. A01 wells sampled from 3 of the 5 independent biological replicates) across all 10 possible combinations of the 5 experiments (Fig. 5A, B). For the 0% and 1% DMSO conditions, mean CV% values across experiments and 60 total combinations remained low for all 6 endpoints, ranging from 1.2–7.8% for developmental endpoints (Fig. 5C and Supplementary Fig. 12A–C) and 5.2–17.0% for embryo-related endpoints (Fig. 5D and Supplementary Fig. 12D–F). All values were substantially below the 30% threshold considered acceptable under OECD Test Guideline #222 for earthworms^37,54^, supporting high inter-experimental repeatability of the vivoDART assay. We selected 1% DMSO as the solvent concentration (vehicle control) for subsequent chemical testing because it allows higher chemical exposure concentrations while modestly increasing membrane permeability, which can aid chemical uptake^12,55,56^.

### Statistical power of DART assay parameters

**Fig. 6:**
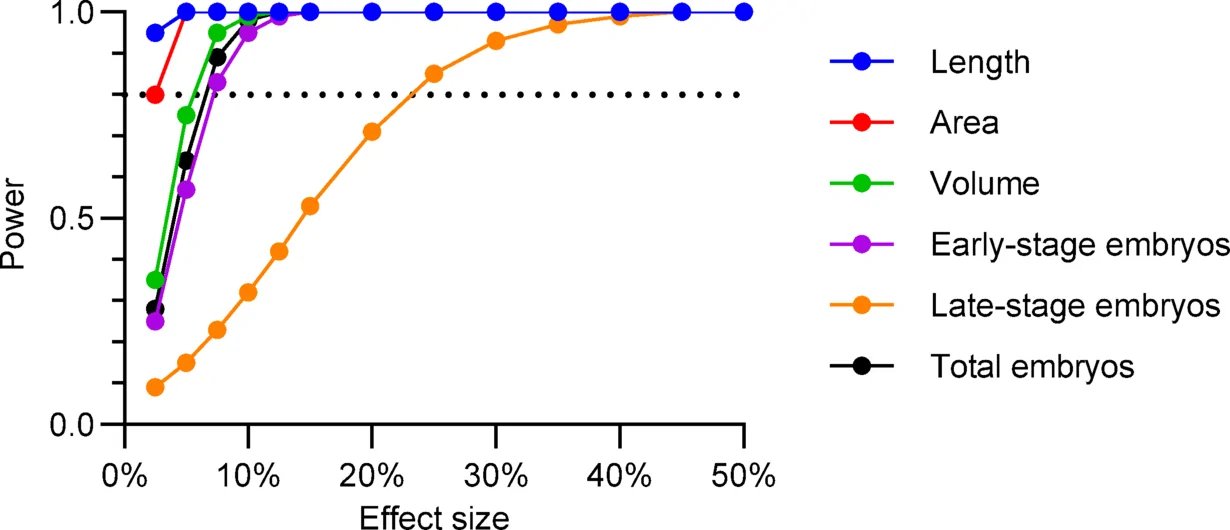
Statistical power for six DART parameters using 3 biological replicates. Predicted statistical power of the assay to detect different hypothetical changes in each phenotype relative to the control (1% DMSO). Power calculations assume 3 biological replicates per condition, the mean and standard deviation measured from all 60 combinations of 3-replicate average values in control populations, and a one-sided significance level of α = 0.05. The dotted horizontal line indicates the 80% power threshold considered acceptable for a good assay performance.

To determine whether the DART assay design had sufficient statistical power of detecting small proportional changes relative to control populations using 3 biological replicates, we calculated the minimum detectable effect sizes required to achieve ≥ 80% power. Power estimates used the aggregated means and standard deviations from all 60 possible combinations (10 combinations per well location and 6 wells) of 3 replicate average values from the 1% DMSO treatment condition which served as the vehicle control. With 3 biological replicates, the assay achieved > 80% power to detect changes of ≥ 5.5% in developmental endpoints (body length, area, and volume), ≥ 7% in total and early-stage embryo numbers, and ≥ 23% in late-stage embryo numbers (Fig. 6A). Increasing the number of biological replicates improved statistical power for all parameters. For example, with 4 and 5 biological replicates, the minimum detectable effect size for late-stage embryo number decreased to 14% and 11%, respectively (Supplementary Fig. 13).

### DART assay with methylmercury

We next evaluated the assay using methylmercury, a well-studied toxicant, with documented toxicity in *C. elegans*^20,25,57^ and mammalian models^58^. The purpose of this experiment was to assess repeatability and precision in concentration–response studies, and to determine the relative sensitivity of each DART endpoint. We ran 3 biological replicates spanning 20 concentrations of methylmercury between 0.0007 and 10 µM using the vivoChip-24x-D1 device.

Range-finding assays based on plate imaging showed that concentrations > 10 µM caused developmental arrest between the L1 and L3 stages, below the minimum worm size that can be trapped in the D1 or L4 devices. Consistently, worms exposed to ≥ 8 µM developed slowly, remained approximately at the L4-stage, and slipped through the D1 chip channels because of their small size (Supplementary Fig. 14). To enable imaging at higher concentrations, we ran a second set of experiments using the L4 devices, which can trap smaller worms down to early L4 stage. For the full concentration-dependent DART study, we used data from the D1 chip for lower concentrations and only used the data from L4 chips for the highest concentrations to complete concentration–response curves. Inclusion of the L4 chip data captured the worm populations with small body sizes, contributing to the data near the EC_50_ values for developmental endpoints and up to EC_100_ values for embryo-related endpoints.

**Fig. 7:**
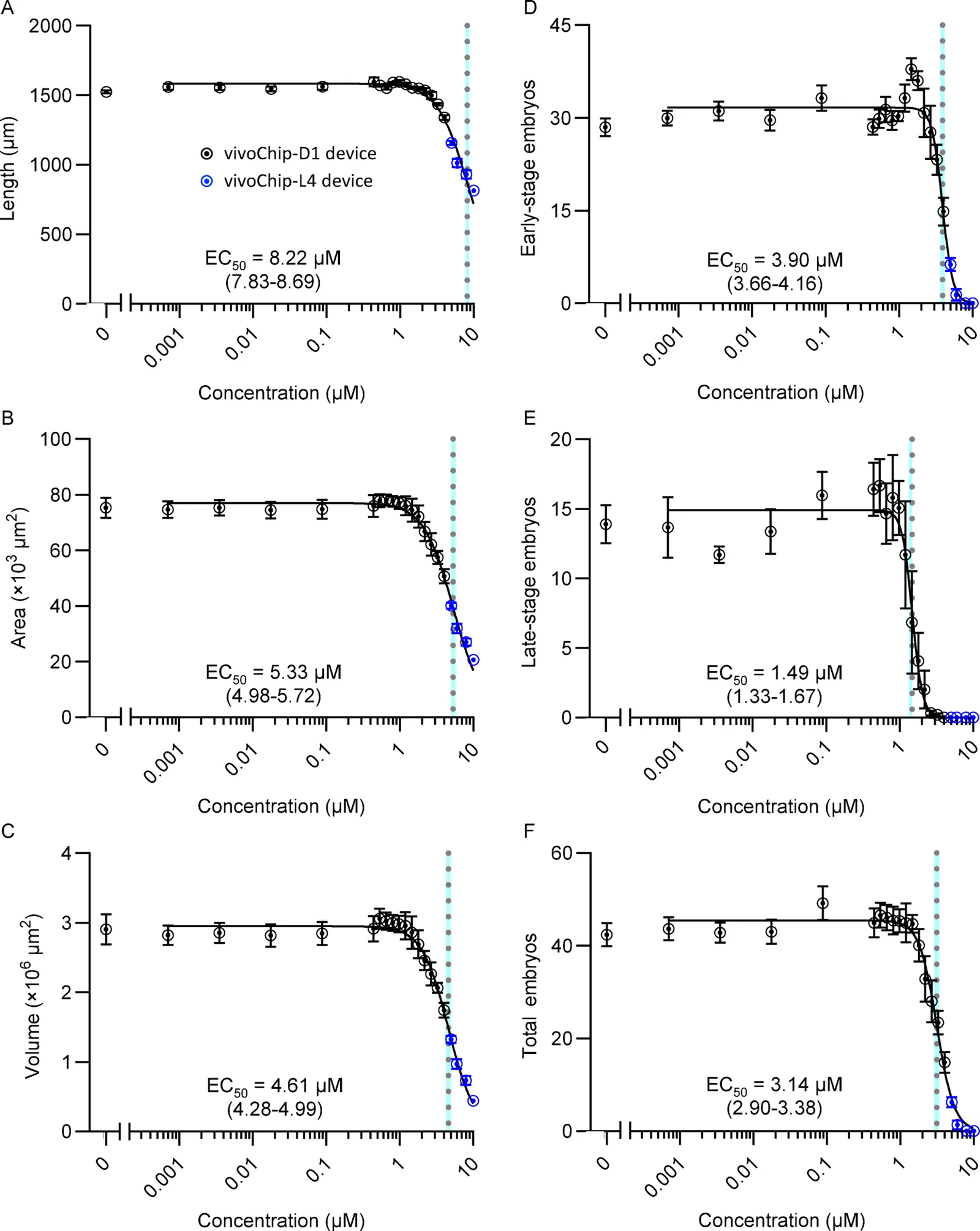
Concentration–response curves of six DART parameters following methylmercury exposure. The effects of methylmercury on (**A**) body length, (**B**) body area, (**C**) body volume, (**D**) number of early-stage embryos, (**E**) number of late-stage embryos, and (**F**) number of total embryos per worm. Data for 0–4 µM methylmercury were obtained from 3 biological replicates performed using the D1 vivoChip devices (black symbols). Data for 5–10 µM methylmercury were obtained from 3 separate experiments performed using the L4 vivoChip devices (blue symbols), as worms at these concentrations were too small to be reliably trapped in the D1 devices. The combined well average values from both datasets were fitted with a 4-parameter logistic curve (solid black lines). EC_50_ estimates are indicated with dotted lines, and corresponding 95% confidence interval bands are shaded in light blue.

The combined results from the D1 and L4 devices were fitted with a 4-parameter logistic curve using well averages after outlier filtering, and EC_10_, EC_25_, and EC_50_ values were estimated for each endpoint (Fig. 7 and Supplementary Table 4). For developmental endpoints, EC_50_ values with 95% confidence interval (CI) were 8.22 µM (7.83–8.69) for body length (Fig. 7A), 5.33 µM (4.98–5.72) for body area (Fig. 7B), and 4.61 µM (4.28–4.99) for body volume (Fig. 7C), indicating that body volume was the most sensitive developmental metric. For the reproductive parameters, EC_50_ values (95% CI) were 3.90 µM (3.66–4.16) for early-stage embryo numbers (Fig. 7D), 1.49 µM (1.33–1.67) for late-stage embryo numbers (Fig. 7E), and 3.14 µM (2.90–3.38) for total embryo numbers (Fig. 7F). These results indicate that embryo classification provides additional sensitivity and that late-stage embryo number is the most sensitive reproductive phenotype. Representative worm images are shown in Supplementary Fig. 15.

Inter-experimental repeatability determined from the 1% DMSO control wells, was high, with CV% values of 1.2% (length), 8.3% (area), 12.9% (volume), 8.8% (early-stage embryo numbers), 17.1% (late-stage embryo numbers), and 10.2% (total embryo numbers). All values were well below the 30% threshold. In addition, the EC_50_ CIs were narrow (< ± 0.5 µM), further supporting assay precision.

To evaluate whether fewer treatment conditions could still support reliable EC_50_ estimation, we omitted every other methylmercury concentration and reanalyzed the DART data using 10 of the original 20 concentrations. We fitted the reduced 10-point dataset with a 4-parameter logistic curve and re-estimated EC_10_, EC_25_, and EC_50_ values (Supplementary Table 5). The resulting EC_50_ values were not significantly different from those obtained using the full dataset for the most sensitive developmental endpoint (body volume; EC_50_ = 4.51 µM; 95% CI 4.03–5.08, *p*-value = 0.98, Supplementary Fig. 16A) and the most sensitive reproductive endpoint (late-stage embryos; EC_50_ = 1.51 µM; 95% CI 1.26–1.80, *p*-value = 1.00, Supplementary Fig. 16B).

To identify a suitable methylmercury concentration of as a positive control for our DART assay, we calculated the strictly standardized mean difference (SSMD) for all 20 concentrations tested and all 6 DART parameters analyzed (Supplementary Table 6). SSMD provides a quantitative measure of assay quality by evaluating the separation between control and treated populations relative to variability. At 4 µM methylmercury, which produced a clear reduction in both developmental and reproductive parameters (Supplementary Fig. 14), SSMD values were: 5.18 (length), 3.24 (area), 2.77 (volume), 2.96 (early-stage embryos), 5.84 (late-stage embryos), and 4.73 (total embryos). According to established criteria, SSMD values ≥ 3 indicate excellent assay quality, whereas values between 2 and 3 indicate good assay quality^59^. Therefore, 4 µM methylmercury demonstrated good-to-excellent performance across all endpoints and was selected as the positive control concentration for the subsequent case study.

Based on the methylmercury concentration–response data, we refined the DART assay design to test new chemicals using 10 concentrations per curve. This approach captured concentration-dependent effects across endpoints with EC_50_ values that differed by up to tenfold. Inclusion of sufficient concentration points is particularly important for endpoints with steep response slopes, such as late-stage embryo numbers, the most sensitive parameter of this assay, to enable precise EC_50_ estimation with narrow 95% CIs.

### Case study: DART assessment of an agrichemical

Following demonstration of assay precision and repeatability, we performed a concentration–response DART study using the triazole fungicide propiconazole^60–62^, a chemical of interest to the agrichemical industry and ecotoxicology. First, we performed a preliminary range-finding assay by treating synchronized L1-stage N2 worms with 22 different concentrations of propiconazole ranging between 0.02–1,730 µM following our standard protocols. Concentrations > 334 µM were lethal (Supplementary Fig. 17).

Based on these results, we conducted a full concentration–response study with 10 propiconazole concentrations (0.08–334 µM), along with the solvent control (1% DMSO), and the positive control (4 µM methylmercury). Using 10 test concentrations and 2 controls (12 wells in total), concentration–response curves for 2 different chemicals can be tested on a single vivoChip-24x device. At the highest propiconazole concentration (334 µM), worms exhibited delayed growth and were immobilized using the L4 chip.

**Fig. 8:**
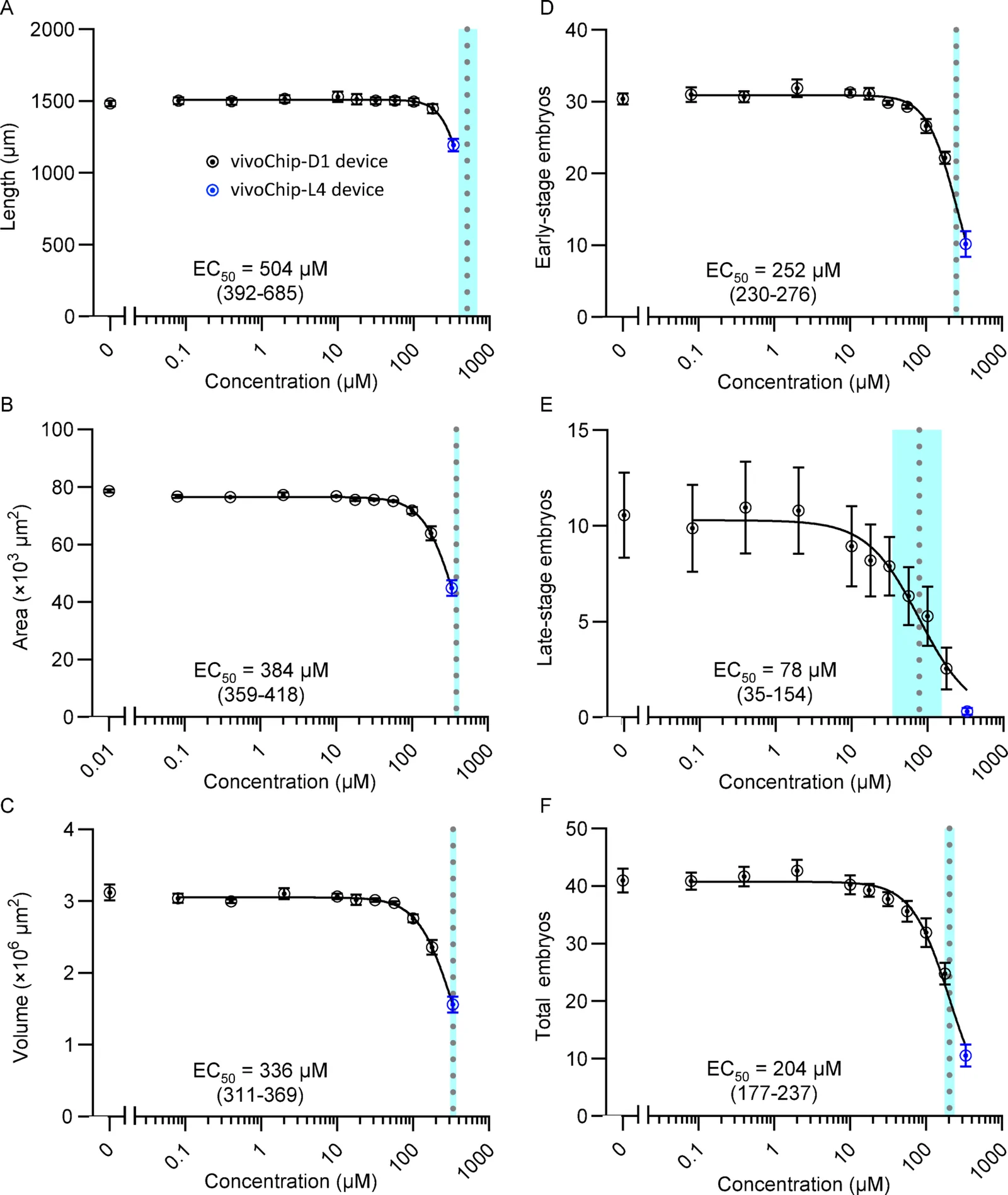
Concentration–response curves for DART parameters following propiconazole exposure. The effects of propiconazole on (**A**) body length, (**B**) body area, (**C**) body volume, (**D**) number of early-stage embryos, (**E**) number of late-stage embryos, and (**F**) number of total embryos per worm. Data for 0–178 µM propiconazole were obtained from 3 biological replicates performed using the D1 vivoChip devices (black symbols). Data for 334 µM propiconazole were obtained from 3 separate experiments performed using the L4 vivoChips (blue symbols), as worms at this concentration were too small to be reliably trapped in the D1 devices. The concentration–response curves were fitted to the combined well-average values from both datasets with a 4-parameter logistic curve (solid black lines). The EC_50_ estimates are indicated with dotted lines, and corresponding 95% confidence interval bands are shaded in light blue.

Similar to methylmercury, propiconazole produced distinct concentration-dependent effects (EC_50_ responses) across developmental (Fig. 8A–C) and reproductive (Fig. 8D–F) endpoints. Among developmental parameters, body volume exhibited a greater sensitivity (EC_50_ = 336 µM; 95% CI 311–369; Fig. 8C) compared to body length (EC_50_ = 504 µM; 95% CI 392–685; Fig. 8A). Among reproductive endpoints, late-stage embryo number was the most sensitive metric (EC_50_ = 78 µM; 95% CI 35–154; Fig. 8E) less than half the EC_50_ for total embryo number (EC_50_ = 204 µM; 95% CI 177–237; Fig. 8F). These results further support late-stage embryo number as the most sensitive DART endpoint in this assay. The EC_10_, EC_25_, and EC_50_ values for all 6 DART parameters are listed in the Supplementary Table 7, and representative worm images are shown in Supplementary Fig. 18.

### Comparative endpoint sensitivity analysis for the DART assay

**Fig. 9:**
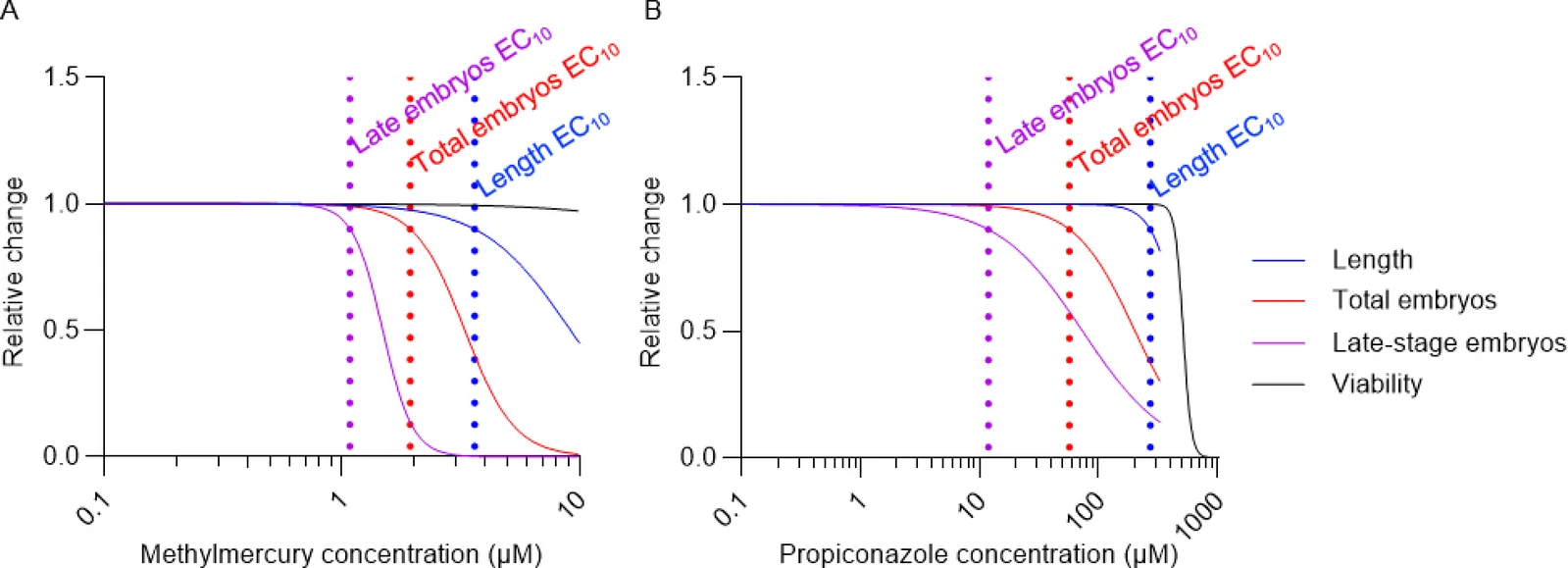
Comparison of normalized concentration–response curves for selected DART endpoints. (**A** and **B**) Concentration–response curves were normalized relative to the control averages for each biological replicate. Four phenotypes are shown for methylmercury (**A**) and propiconazole (**B**): body length, number of total embryos, number of late-stage embryos, and percentage of viable worms. Viability was estimated as the percentage of motile worms based on 10-s movies in culture plate wells. Estimated EC_10_ concentrations are indicated for body length, total embryo number, and late-stage embryo number. The leftward shift of the late-stage embryo curve relative to the other sub-lethal endpoints, together with its separation from viability loss, demonstrates that this endpoint is the most sensitive among the DART-related endpoints and is independent of lethality.

To further evaluate the relative sensitivity and utility of different DART endpoints, we compared normalized concentration–response curves for a commonly used developmental parameter (worm body length), and two reproductive parameters, total and late-stage embryo numbers, for both methylmercury (Fig. 9A) and propiconazole (Fig. 9B). Concentration–response data for all parameters were normalized to the vehicle values. In addition, we quantified the percentage of viable worms based on motility in culture well plate movies captured using a 2× objective.

Worm viability remained high across the concentration ranges used in the vivoChip concentration–response experiments. For example, 100% of worms remained viable at 334 µM propiconazole and more than 97% remained viable at 10 µM methylmercury, despite clear developmental impairment. These findings indicate that the observed responses were attributable to DART-specific effects rather than lethality, a non-specific apical endpoint.

We calculated EC_10_ values for body length, total embryo number, and late-stage embryo number for both methylmercury and propiconazole using the normalized concentration–response data (Fig. 9, Supplementary Tables 4 and 7). The EC_10_ estimates, representing the onset of detectable toxicity for each endpoint, were clearly separated and followed the same sensitivity order for both chemicals: late-stage embryo number < total embryo number < body length < lethality onset. For methylmercury, the EC_10_ values were 3.62 µM (95% CI 3.33–3.92) for body length, 1.94 µM (95% CI 1.71–2.19) for total embryo number, and 1.08 µM (95% CI 0.92–1.24) for late-stage embryo number. For propiconazole, the EC_10_ values were 273 µM (95% CI 237–308) for body length, 57 µM (95% CI 43–73) for total embryo number, and 12 µM (95% CI 6–21) for late-stage embryo number. Notably, the EC_10_ for late-stage embryo number preceded any measurable response in the other DART endpoints, identifying late-stage embryo number as the most sensitive parameter.

For both methylmercury and propiconazole, the EC_10_ value for body length, a commonly used developmental endpoint, was significantly higher than the EC_10_ for late-stage embryo number, the most sensitive reproductive endpoint. Specifically, body-length EC_10_ values were 3.4 × higher for methylmercury and 22.8 × higher for propiconazole than the corresponding late-stage embryo EC_10_ values, with both comparisons reaching statistical significance (*p*-value < 0.001). Viability remained high across the tested concentration ranges, confirming that the observed changes in each DART parameter represented bona fide developmental and reproductive effects rather than secondary consequences of lethality. These findings demonstrate that the multiparametric vivoDART assay can distinguish reproductive toxicity from developmental toxicity and viability loss, providing independent and biologically meaningful assessments of developmental and reproductive endpoints without confounding effects from reduced viability.

This significantly lower toxicity threshold for late-stage embryo number, especially for propiconazole, highlights the added sensitivity of embryo-stage classification compared with conventional developmental endpoints such as body length. This multiparametric approach enables the identification of the earliest adverse-effect onset across biologically distinct endpoints, supporting its utility for chemical prioritization and comparative safety assessment.

## Discussion

DART studies are required for hazard assessment of thousands of new and existing substances. However, high costs, long study durations, and the global push to reduce or eliminate mammalian models in routine testing present major bottlenecks. Here, we introduce vivoDART, a novel imaging-based DART assay using the model organism, *C. elegans*. This platform is fast, cost-effective, and does not utilize vertebrate animals, which are subject to welfare regulations.

*C. elegans* hermaphrodites have a complete reproductive system with molecular pathways and several hallmarks of reproductive aging conserved with human females^40^. In certain toxicological domains, *C. elegans* have been shown to be as predictive as rodent models^13,28,43^, supporting the translational relevance of data obtained with this system for human reproductive toxicity assessment. While *C. elegans* has been used as a model organism in DART studies, previous approaches were limited by low throughput, labor-intensive methodologies, or reliance on relatively gross phenotypes such as brood size or body length^44,45^. In this work, we developed a high-content, multiparametric imaging platform capable of detecting subtle multi-parametric DART phenotypes. This approach increases assay sensitivity to low-level exposures while improving throughput, thereby addressing key limitations of existing DART testing paradigms.

We previously developed an ML-based imaging platform to assess DevTox using *C. elegans* as an alternative model organism^46^. The present study expands upon that work by incorporating reproductive toxicity-related endpoints to assess multiple modes of action within a unified DART framework. We leveraged the vivoChip platform, which enables high-resolution, high-throughput imaging of 24 independent worm populations, with up to 40 worms per population (totaling up to 960 worms per vivoChip). The design of microfluidic vivoChip generates high-information-density datasets suitable for quantitative assessment of in utero embryo production and developmental progression. Two different vivoChip-24x designs, each optimized for trapping worms of different sizes, allowed us to analyze DART endpoint changes ranging from delayed L4 stage worms to normally developed adults. Integrating data obtained from both device configurations enabled comprehensive coverage across different sections of the concentration–response curve. This capability is particularly essential for multi-parametric assays, in which EC_50_ values for distinct endpoints may be widely separated, and therefore require adequate sampling across multiple exposure ranges to accurately resolve differential sensitivities.

For our vivoDART assay, we exposed age-synchronized L1 stage worms for 72 h and performed endpoint imaging of *C. elegans* populations using the vivoChip platform. Worms were treated with multiple conditions, including solvent controls, in which worms developed to the D1 adult stage during the exposure period. Because toxic chemical exposures can cause slow and asynchronous development, especially at high concentrations, it was necessary to start experiments with precisely age-synchronized worms. Reproductive organ development in *C. elegans* spans from the L1 stage to early adulthood and includes cell division of the two somatic gonad precursors and two primordial germ cells at the L1 stage, reorganization of the somatic gonad primordium during late L2, and formation of the sheath, spermatheca, and uterus during the L3 and L4 stages^41^. A 72-h chronic exposure starting at the L1 stage maximizes the likelihood that chemicals reach the germline during critical windows of development. Such exposure may interfere with key biological events, such as mitotic proliferation, meiotic cell cycles, spermatogenesis, oogenesis, oocyte maturation, ovulation, and the entire course of in utero embryogenesis up to the start of neuromuscular movement within the egg^63,64^. Imaging all treatment groups within a narrow time interval further ensured accurate comparisons across concentrations and minimized variability introduced by differences in developmental timing.

To capture adverse effects on reproduction, we identified and quantified all embryos within the uterus of individual *C. elegans* hermaphrodites and classified them as early- or late-stage embryos based on distinct morphological features, using the twofold stage as the classification threshold. We also calculated total embryo numbers by adding early- and late-stage embryo numbers for each worm. While the total embryo number serves as an in utero proxy for the traditional brood-size metric, the late-stage embryo numbers provide an indicator of successful embryonic development and is conceptually related to the hatching efficiency measurements. Compared to traditional reproductive toxicity assays, vivoDART offers several advantages, including (1) scalability enabled by well-plate-compatible liquid culture, (2) simultaneous testing of 24 independent populations, (3) high-resolution imaging of up to 40 *C. elegans* per population to quantify in utero embryonic development, and (4) single time point imaging that reduces assay complexity while preserving multiparametric information content.

Traditional brood size assays performed on NGM plates are labor-intensive and low-throughput^44^. Although microfluidic-based automated egg counting systems have been developed^49–51^, these platforms are technically complex, require continuous imaging over 3–6 days, and consume large amounts of test substances due to constant perfusion. In contrast, vivoDART assay quantifies and classifies all in utero embryos within hermaphrodites on the first day of adulthood, when embryo production enters the peak phase. Assessing DART endpoints at this early adult stage offers several key advantages: (1) it enables scoring of high-quality embryos while avoiding confounding effects from age-related gonadal atrophy; (2) embryos are typically aligned in a single row, reducing phenotyping errors that arise from multilayered embryo stacking in older worms; (3) the readout is independent of the egg-laying circuit, minimizing data drift caused by off-target neuromuscular effects; and (4) the assay is based on direct morphological features of embryogenesis rather than late-stage neuromuscular strength, which influences larval hatching outcomes. In addition, vivoDART assay relies on brightfield image analysis of wild-type worms and does not require reporter strains or fluorescent biomarkers. This feature facilitates application of the assay to large numbers of wild isolates with diverse genetic backgrounds, enabling characterization of genetic susceptibility to environmental toxicants using naturally occurring variation in *C. elegans* populations^22^.

For any newly developed method, intra-laboratory repeatability (and ultimately inter-laboratory reproducibility) is vital for consideration by regulatory bodies^10,65^. To address repeatability, we established robust and detailed protocols for *C. elegans* husbandry, synchronization, chemical exposure, and culture conditions to minimize inter-experimental variability. The vivoDART assay is highly data-intensive, generating ~ 11,000 images and nearly ~ 325 GB of data in less than 30 min from a single experiment. To enable even higher throughput, we developed a minimal data acquisition protocol that reduces data volume while preserving the ability to produce all DART data presented in this study. This optimized approach, suitable for large-scale studies, collects 2,184 images (~ 66 GB) from a single vivoChip by limiting imaging to a single timepoint, 18 brightfield z-slices. To manage such large volumes of data and support multiparametric phenotypic analysis, we developed the vivoScreen platform, which integrates reliable hardware components with rigorously validated and tested software, centralized database infrastructure, and both local and cloud-based data storage systems. Every experiment is archived with comprehensive metadata, including experimental details, imaging conditions, analysis outputs, and final reports, ensuring full traceability. Despite the large data volume, image analysis is performed efficiently using an ML-based pipeline for DevTox parameters with accuracy comparable to human scorers while requiring only a fraction of the time. Embryo phenotypes were then scored using semi-automated, user-friendly software designed to streamline manual embryo annotation and improve scoring consistency.

The mean CV% for 1% DMSO ranged from 1 to 5% for developmental endpoints and 6–17% for embryo-related endpoints, values well below thresholds considered acceptable for regulatory-approved assays, including those described for earthworms^37^. This low variability confers high statistical power, even with relatively few experimental replicates. Power analysis indicated that 3 independent experimental replicates were sufficient to detect changes > 23% across all endpoints, while smaller effect sizes could be reliably identified with additional replicates. For late-stage embryos, the most variable DART endpoint, detectable effect sizes improved from 23 to 14% and 11% using four and five biological replicates, respectively. While the late-stage embryo phenotype exhibited the highest variability, it was also the most sensitive endpoint in terms of EC_50_. The higher variability likely reflects the oscillatory pattern of egg laying, as embryos are laid in clusters every 1–2 h^66^. Depending on timing, worms may be immobilized and imaged immediately before or after an egg-laying event, introducing natural biological variability into embryo counts. This effect was especially pronounced for the late-stage embryos parameter, where the small number of late-stage embryos caused the CV% to increase. Among the four DMSO conditions, variability in early-stage and total embryo numbers were higher in water-control wells but decreased in DMSO-treated wells. In addition, we confirmed that 1% DMSO, used as the vehicle control, did not contribute to any significant adverse effects on embryo-related endpoints. This aspect will enable testing of many chemicals at relatively wide treatment concentrations^55,56^.

We validated the vivoDART assay using two reference chemicals: methylmercury and propiconazole. Methylmercury was chosen because it has been extensively evaluated across variety of species and toxicology domains, including DART, neurotoxicity, metabolism, gene expression, stress response, and cell division^20,25,67–70^. Propiconazole was chosen because it is a widely used fungicide with well-characterized toxicological profiles in mammals and other model organisms^60,61,71,72^. Both chemicals showed concentration-dependent effects across all 6 endpoints studied in the vivoDART assay. These endpoints exhibited differential sensitivities based on EC_50_ values, with body volume emerging as the most sensitive developmental parameter and late-stage embryo numbers as the most sensitive reproductive parameter. We observed narrow 95% confidence intervals and low CV% values for the methylmercury control wells, indicating high assay precision. The assay quality, measured using SSMD, was good to excellent for ≥ 4 µM methylmercury, supporting its suitability as a positive assay control for all endpoints.

The onset of DART toxicity for methylmercury in our assay, based on the late-stage embryo endpoint, occurred at ~ 1 µM, consistent with previously reported *C. elegans* DART studies^20^. For propiconazole, adverse effects on fathead minnow fecundity and *Daphnia* embryonic development have been reported at 1.46 µM (0.5 mg/L), which is within an order of magnitude of our EC_10_ value of 14 µM^71,72^. A recent study in zebrafish reported a DART EC_50_ of a 1.62 mg/L, (~ 2 µM propiconazole equivalent) for a commercial formulation, and additional studies have observed developmental abnormalities at 0.73–1 µM^73–75^. Differences across species may reflect differences in chemical uptake, accumulation, or metabolism, which could be addressed in future studies by measuring tissue-level concentrations using analytical methods. In *C. elegans*, previously reported developmental toxicity and brood-size-based reproductive toxicity assays reported EC_50_ values of 223–249 µM and 141 µM, respectively^76,77^. In comparison, the EC_50_ value for the most sensitive late-stage embryo parameter in our assay was 78 µM, ~ 1.8–3.2× lower than these previously reported values. These differences could be associated with differences in food source, DMSO concentration, exposure duration, and endpoint definition. While the absolute effective concentrations of propiconazole in our results are higher than those reported in vertebrate systems, the *C. elegans*-based vivoDART platform offers substantial advantages in cost, scalability, and throughput. By testing two reference chemicals, we demonstrated a broad dynamic range in concentration–response data with EC_10_ values spanning 1.0 – 237 µM. Such a large dynamic range is valuable because it enables distinguishing of subtle biological effects from stronger toxic responses across a broad exposure window, supporting sensitivity, reproducibility, mechanistic interpretation, and confidence in benchmark concentration estimates. Compared with rodent colonies and fish aquaria, this approach requires substantially fewer resources and eliminates labor-intensive and subjective phenotyping of complex reproductive phenotypes on an animal-by-animal basis. Moreover, its imaging-based multiparametric design enables objective and quantitative assessment of developmental and reproductive phenotypes.

All endpoints studied in the vivoDART assay are biologically interlinked, and reproductive (embryo) phenotypes may, in part, be affected by developmental perturbations. However, effective concentrations for different endpoints were clearly separated, suggesting distinct endpoint sensitivities rather than a uniform secondary response. Total embryo numbers declined at lower concentrations than those required to reduce body size, indicating that the germline was specifically affected and that the observed reproductive effects were not simply a consequence of delayed general development. Moreover, reductions in late-stage embryos occurred before a decrease in total embryo numbers, consistent with direct impairment of embryonic development rather than a simple reduction in embryo production. Importantly, the worms remained > 97% viable and motile across all tested concentrations. Therefore, the measured effects reflect DART-specific responses rather than non-specific apical endpoints such as lethality.

The vivoDART platform, presented here, is both faster and more cost-effective than traditional vertebrate-based assays. Thanks to the short life cycle of *C. elegans*, culture and treatment durations are measured in days, while chip loading and imaging times require < 30 min per experiment, improving efficiency. Data analysis can also be performed rapidly. The ML inference pipeline used for body-dimension quantification can process a single chip (~ 1,000 *C. elegans*) in < 5 min using a standard desktop computer. Across these studies, we collected and annotated data from over ~ 389,000 embryos across ~ 9,200 worms under various treatment conditions, including detailed classification of developmental stage and extraction of *xyz* centroid coordinates within image stacks. This extensive dataset provided a strong foundation for training next-generation ML models to fully automate embryo detection and staging. Since completion of the present study, an ML-accelerated image-analysis pipeline for automated embryo phenotyping has been successfully implemented and validated, and those results will be reported in a separate manuscript. Implementation of a fully automated analysis pipeline supports future adoption of a dynamic testing strategy in which an initial range-finding assay is first performed using widely spaced concentrations, followed by endpoint-specific concentration refinement to better characterize changes in response slopes and improve EC_50_ estimation. Such an approach would reduce experimental burden and allow more chemicals to be tested on each chip, increasing assay efficiency and scalability.

This study demonstrates the sensitivity, repeatability, and precision of the vivoDART assay. Automated embryo scoring will further enable high-throughput screening of large number of reference chemical libraries to establish assay specificity and sensitivity across defined chemical spaces. These advances support vivoDART as a rapid and cost-effective whole-organism testing platform for chemical prioritization and comparative hazard assessment.

## Materials and methods

### Strains and maintenance

We used the N2 wild type strain (The *Caenorhabditis* Natural Diversity Resource, CaeNDR) for all experiments. *C. elegans* were maintained according to standard methods on nematode growth media (NGM) agar plates seeded with HB101 *Escherichia coli* bacteria at 20 °C^78^. Frozen stocks of strains were stored at − 80 °C and thawed annually to generate a new set of Dauer plates. Sealed Dauer plates were stored at 16 °C and used to start fresh maintenance cultures every 3 months by chunking, minimizing genetic drift. We cultured worms for > 5 generations with excess food before using them in assays in a temperature and humidity-controlled environment.

### Food preparation

We streaked HB101 from − 80 °C glycerol stock onto Luria–Bertani (LB) agar plates with streptomycin and incubated them overnight at 37 °C to generate single colonies. A single colony was picked and used to inoculate an overnight 5 mL starter culture in LB broth containing streptomycin. To prepare the HB101 food stock, 200 µl of this starter culture was added to 200 mL LB broth and streptomycin in a 1,000 mL baffled flask. The flask was incubated for 16 h with shaking at 180 rotations per min (rpm) to reach the late log growth phase. We measured the OD_600_ using a spectrophotometer (DiluPhotometer™, Implen) and stored the culture at 4°C. Streak plates and starter or food stocks were used within 2 months and 2 weeks, respectively. Immediately before worm culture, we centrifuged a sufficient volume of the bacterial culture to generate the required volume of OD_600_ = 3.0 food suspension. The LB supernatant was removed, and the bacterial pellet was resuspended in S media. This density was optimal for growing 80–100 larval 1 (L1) to the day 1 (D1) adult stage in a 500 µl culture volume, with sufficient food remaining after 72 h to avoid starvation in the worm population prior to DART analysis. The D1 adult worms are defined as worms reaching early adulthood with ~ 40 embryos within 72 h post L1 feeding growing at 20 °C in the vehicle control populations.

### Chemicals

Methylmercury (II) hydroxide (Cat# 13395, Alfa Aesar, CAS# 1184-57-2, Batch# M25H004) and propiconazole (Cat# 45642-250MG, Sigma Aldrich, CAS# 60207-90-1, Batch# BCCD0480) were dissolved in dimethyl sulfoxide (DMSO, Cat# D2650, Sigma Aldrich, CAS# 67-68-5, Batch# RNBL9635) to prepare master stocks at 100 × the highest treatment concentration. The master stocks were transferred into 0.2 ml tubes as single-use aliquots and kept at − 80 °C until use. For the concentration–response assays, chemicals were serially diluted in DMSO immediately prior to treatment to generate 100× working dilutions, ensuing a final DMSO concentration of 1.0% (v/v) in each well. Solvent control wells received DMSO at the same final concentration (1.0% v/v).

### Worm synchronization and large-scale culture

To generate large numbers of synchronized L1-stage larvae, two NGM plates were each seeded with four parent larval 4 (P_0_ L4) worms. The worms were grown for 48 h to allow P_0_ to become adults and lay a large number of eggs. At this time, all 8 P_0_ adults (2 NGM plates × 4 adults/plate) were removed. The plates were incubated for an additional 48 h, by which point the majority of F_1_ generation had reached adulthood. Adult F_1_ worms were collected by washing both plates with M9 buffer and treated with an alkaline sodium hypochlorite solution with alternating low- and high-speed shaking on a speed-controllable vortexer to fragment their bodies and release embryos. The bleaching solution was neutralized with 5 washes of M9 buffer. Embryos were allowed to develop and hatch in M9 buffer in a rotating glass conical tube for 24 h. Hatched synchronized L1s were filtered through a 20 µm cell filter to remove unhatched eggs and debris. The total number of larvae was counted, and the suspension volume was adjusted to make a final density of ~ 100 L1s/20 µl of M9 buffer.

### Chemical treatment in liquid culture

The age-synchronized L1 suspension (~ 100 larvae in 20 µl volumes) was dispensed into individual wells of a standard 24-well plate with 475 µl HB101 suspension (OD_600_ = 3.0) prepared in S media^79^. Chemicals dissolved in DMSO (5 µl) were added to the designated wells to achieve the desired treatment conditions while keeping a constant solvent concentration across all wells. Vehicle control wells received 5 µl of DMSO solvent (1% v/v). The 24-well culture plates were sealed with airtight films (Cat# 232702, Thermo Scientific) to prevent evaporation and cross-contamination and cultured at 20 °C for 72 h. The plates were shaken every 6 h using an automated shaking platform (Teleshake 95, Inheco) to homogenize the well contents, including the HB101 distribution, shed cuticles from molting, and the intestinal discharge from the defecation cycles.

### Range-finding assay and live worm quantification

For each chemical tested, we first performed a range-finding assay using at least 10 concentrations (0.009–90 µM for methylmercury and 0.01734–1730 µM for propiconazole) based on published work. Age-synchronized L1s were exposed to different chemical concentrations for 72 h to determine the maximum concentration in culture medium and assess any significant lethality or growth arrest at the L1 stage. We manually inspected the well-plate images, particularly wells containing worms analyzed for sublethal developmental or reproductive toxicity phenotypes, to confirm that no chemical precipitation occurred during culture. Age-synchronized L1s were exposed to these concentrations in 24-well plates for 72 h, after which culture plates were imaged and manually analyzed prior to chip transfer. The plate images were used to identify the percentage of live worms and to identify the highest concentration at which the worms developed beyond the L4 stage, ensuring suitability for subsequent vivoChip loading and high-resolution imaging for DART phenotyping.

Specifically, prior to chip imaging, a single-frame and timelapse images (10 s at 10 fps with a 2× , 0.08 NA objective) were acquired from each 24-well culture plate using the vivoScreen system (described below), generating ~ 10.8 GB of data per plate. Single-frame images were inspected to ensure that control wells developed to D1 adult stage according to our predefined assay acceptance criteria (Supplementary Table 6). Time-lapse images were inspected to count all worms within the 10,744 × 6,290 µm^2^ field of view (FOV), and spontaneous movement (motility) in liquid culture was used as the criterion for viability. The viability percentage per well was then calculated from 3 biological replicates on different days.

### vivoChip-24x design and operation

Microfluidic-based *C. elegans* immobilization chips (vivoChip-24x, vivoVerse), fabricated from a transparent, low-chemical-absorptivity plastic with broad optical transmission spectrum^21^, were used in this study. The device consists of three bonded layers: (i) a top layer containing 24 rectangular wells arranged with 9 mm spacing for compatibility with standard 24-well plates, (ii) an intermediate microfluidic layer containing trapping channels, and (iii) an ultra-thin ~ 80 µm bottom substrate layer enabling high-resolution optical access.

Each well has a loading capacity of 250 µL and is fluidically connected at its bottom surface to an array of 40 parallel tapering microfluidic channels, as previously described^46,47^. In total, the chip contains 960 channels that orient and immobilize ~ 40 *C. elegans* per well, enabling rapid, high-resolution imaging of 24 distinct worm populations. The channels possess a 3D tapering geometry with an aspect ratio (width to height ratio) close to 1.0, promoting lateral worm orientation. Gradual reduction in channel dimensions allows size-dependent trapping along defined channel regions.

Two vivoChip-24x designs were used: the vivoChip-24x-D1 design with larger channel geometry (tapering from 98 μm × 102 μm at the channel entrance to 24 μm × 40 μm at the exit) and vivoChip-24x-L4 design based on narrower geometry (tapering further to 10 μm × 17 μm at the narrowest region). The D1 design is optimized to trap day 1 (D1) adult worms containing 2–6 in utero embryos at the narrowest channel region while allowing laid eggs to pass through. The L4 design enables trapping smaller worms, including early L4 stage worms, and is better suited for populations with fewer or no embryos, although it carries a higher risk of blockage from laid eggs and clogging the microfluidic channels.

Accordingly, two functional designs were used: the vivoChip-24x-D1 (based on the larger channel geometry) and the vivoChip-24x-L4 (based on the narrower geometry). The D1 chip was preferentially used because its wider channels prevented blockage by laid eggs and therefore had a greater number of channels containing trapped worms. The L4 chip was mainly used for worms treated with high concentrations of chemicals that slowed development, resulting in reduced body size and fewer or no in utero embryos.

Following removal of the culture plate seal, worms were transferred from each culture plate to the corresponding vivoChip well using a multichannel pipette. Since vivoChip-24x-L4 can trap L4-stage or larger worms, wells containing populations younger than L4 were filled with molten 2% agarose gel to block the channels in those specific wells. The agar block prevented continuous media flow through the open channels and stabilized pressure across all wells during vivoChip imaging.

The chip was placed in a custom vivoChip holder with a sealing gasket to provide controlled fluidic pressurization, as previously described^22^. The vivoChip holder carrying the vivoChip was placed on the microscope stage for imaging and flow was initiated using our vivoCube + microfluidic control system. Ten on/off pressure cycles were applied, resulting in complete immobilization of *C. elegans* across all 960 channels within ~ 3 min.

### Automated image acquisition hardware

The vivoScreen system comprises integrated image acquisition hardware, custom software modules (vivoImager), a centralized searchable Postgres SQL database, and hybrid in-house and cloud-based data storage (Supplementary Fig. 2). The image acquisition hardware consists of a customized large FOV inverted microscope (IX73, Evident), a precision *xyz* motorized stage (MS2000, Applied Scientific Instrumentation), a scientific CMOS camera (IRIS-15, Teledyne), and bright light sources (TLED, Sutter Instrument). The large-FOV setup enables imaging of the entire chip area with a 2× , 0.08 NA objective and 8 microfluidic channels simultaneously using a 10× , 0.4 NA objective, capturing all 40 channels with only 5 FOVs. The 10× objective produces a 2.15 × 1.26 mm^2^ FOV, sufficient to capture the full lengths of adult worms across 8 channels with a lateral pixel resolution of 0.425 µm. For DART analysis, volumetric brightfield imaging was performed using 18 z-slices at 6 µm step size to capture the entire worm volume. According to the Nyquist sampling criterion, the theoretical axial resolution for a 0.4 NA objective is 6.5 µm, corresponding to a recommended z-step of 3.25 µm. However, to maintain volumetric imaging speed ~ 1 volume per second with our data readout speed, we used a 6 µm step size. We captured five timelapse 3D hyperstack images at 1-s intervals to analyze twitching in the late-stage embryos if needed.

### Automated image acquisition software and process

To acquire the imaging data, we used custom-developed image acquisition software (vivoImager, Supplementary Fig. 3). We captured 10× , 0.4 NA 3D brightfield stacks (18 z-slices) of all worms, centered around the best focal plane of a fiduciary marker (Supplementary Fig. 19A). Each vivoChip-24x well contains a cross-shaped fiduciary marker, integrated into the microfluidic channel layer. This feature enables automatic focal plane identification using an edge-detection algorithm (Supplementary Fig. 19B). Initially, the software loads a configuration file containing experimental metadata (experiment name, strain name, developmental stage, chemical name, concentration, solvent, etc.) and imaging parameters (illumination intensity, filter sets, camera settings, stage locations, etc.). Then the software initiates all hardware components, calibrates the stage for translation range, and positions the stage at the cross-shaped fiduciary marker of the reference well (B02). Using a pre-defined chip map, the system sequentially navigates to the fiduciary marker locations in each of the 24 wells to determine the best focal plane. Variations in focal position primarily arise from substrate curvature during chip pressurization.

While the relative channel locations are known from the chip map, the positions of the trapped worms within the 40 parallel, 3 mm-long channels may vary depending on their size. To optimally center the 1.3 mm × 2.2 mm FOV to capture the entire body of as many worms as possible, a low-magnification overview image of the entire chip is first captured using a 2× , 0.08 NA objective. Then, all immobilized worms are detected using a trained YOLO object detection model^23^, and the relative stage positions are calculated for the 5 FOVs needed to image the worms at 10× , 0.4 NA (8 × 150 µm spaced channels can fit in each 1.3 mm wide FOV) (Supplementary Fig. 19C). The system then automatically switches to the 10× objective and captures brightfield z-stack images using high-precision 3-axis translational stages. For each well, 5 FOVs are imaged, each comprising 18 z-planes (6 µm step size) and five timepoints in brightfield for volumetric timelapse acquisition (Supplementary Fig. 19D).

A single vivoChip experiment generates 12,984 images (~ 390 GB). Raw image data and associated metadata (timestamps, xyz coordinates, image capture and illumination parameters, treatment conditions etc.) are automatically transferred to a high-capacity network attached storage (NAS) server for preprocessing and downstream analysis.

### Image processing and phenotypic scoring

For each FOV with 8 channels, channel boundaries were identified by detecting fiduciary markers within each well and applying pre-defined internal channel spacing from the chip map. Channel coordinates were stored in the centralized database for subsequent multiparametric analysis.

Automated worm detection was performed using a deep learning–based method^46^. In brief, an image slice with optimal focus was identified from each z-stack by maximizing the variance of the Laplace-transformed image^80,81^. An improved 2.5D U-Net architecture, including a classification head at the bottleneck, was trained, tested, and used to first classify each channel as full (entire worm within the FOV), partial (partial worm within the FOV), or none (no worm present) and then predict a segmentation mask for channels classified as full. For the new model, we expanded the training dataset to include small-sized worms close to the channel exit, decoupled the image-level classification and the pixel-wise segmentation models to facilitate independent optimization and model learning with 1 and 5 z-slice images around the best focus image, respectively. The segmentation mask, predicted by the new model, was used to calculate body length (via skeletonization of the mask), area (total segmented pixels), and volume (mask area integrated with channel heights) for each full worm.

Embryonic phenotyping was performed manually on all channels predicted as “full” by the ML model. All in utero embryos in these channels were scored and classified according to their developmental stage. Ten trained scorers independently scored and classified embryos using an in-house developed graphical user interface (vivoAnalyzer, Supplementary Fig. 8). The software displays individual channels with zoom and z-plane navigation capabilities, allowing users to place a marker on each embryo and classify it as early- or late-stage embryo using the twofold stage as the classification threshold. All automated image processing outputs (worm classification labels and segmentation masks) and manual annotations (marker positions and classifications of each embryo) were stored in the centralized database and exported for statistical analysis. The majority of embryo scoring was conducted in a blinded manner, with treatment conditions concealed from scorers.

#### Statistical analysis

For DART data analysis, datasets were first cleaned to remove any worms for which the body segmentation mask failed, preventing dimensional calculations, or channels marked as unscorable by a user due to visual obstruction of the uterus by the intestine (Supplementary Fig. 9). The remaining worms were then filtered to remove outliers in body volume or total embryo count (sum of early- and late-stage embryos). Outliers were identified using Tukey fences (1.5× the interquartile range (IQR)) applied to each endpoint, and worms with measurements outside these fences were excluded. Inspection of excluded samples indicated that most were debris composed of shed worm cuticle and laid embryos clogging the channel. Mean phenotype scores were calculated per well and used to fit concentration–response curves from 3 biological replicates. One-way ANOVA was used to identify statistical differences between multiple well average values for different DMSO conditions.

To estimate repeatability, the CV%, defined as the ratio of standard deviation to mean, was calculated for every possible combination of 3 wells drawn from every corresponding well position across 5 independent biological replicates (10 combinations per well position, 24 well positions). The mean CV% and standard error of mean (SEM) across all 10 combinations were reported for each endpoint. To measure the strengths of the difference between 1% DMSO and methylmercury-treated worms, we calculated the Strictly Standardized Mean Difference (SSMD) for all 6 endpoints using 3 biological replicates. From all methylmercury conditions, we selected the lowest concentration to serve as a positive control for our case study that produced the SSMD ≥ 2 for all the DART parameters, indicating strong effects^59,82–84^.

Assay power was estimated using the mean and standard deviations obtained from all 60 possible combinations (10 combinations per well position × 6 well positions with 1% DMSO) for all endpoints from repeatability experiments. Power calculations were performed for a fixed value of *n* = 3 technical replicates and a series of hypothetical mean representing 2.5%—50% effect sizes below the control means (one-sample, one-tailed, t test, α = 0.05) using GraphPad Prism (version 10.6.1).

Concentration–response curves and effective concentration (EC_10_, EC_25_, and EC_50_) values were fitted using a 4-parameter logistic curve function using the “Find ECanything” nonlinear fit function of GraphPad Prism. The lower asymptote for each endpoint was constrained to the minimum biologically possible values of 0 embryos for Reproductive endpoints and estimated mean L1 body dimensions (length = 150 µm, area = 1,500 µm^2^, volume = 11,775 µm^3^) for developmental endpoints. For each EC_x_ estimate, the 95% confidence interval (CI) bands were calculated and plotted. To study relative sensitivity among different DART and viability parameters, we normalized the concentration–response values to the vehicle control values in each biological replicate for all parameters. The normalized concentration–response data was fitted using 4-parameter logistic curve function to analyze and compare the EC_10_ values for different parameters. Two concentration–response curves were compared using extra sum-of-square model to identify if the best fit values of EC_x_ values and Hillslope differ between two data sets. The p-value was denoted by ns (*p*-value ≥ 0.5), * (*p*-value ≤ 0.01), ** (*p*-value ≤ 0.001), *** (*p*-value ≤ 0.0001), and **** (*p*-value ≤ 0.0001).

## Supplementary Information

Below is the link to the electronic supplementary material.

Supplementary Material 1

## Data Availability

The datasets generated and/or analyzed during the current study are available from the corresponding authors upon reasonable request.
