# Supplementary material for: A robust, high-content NAM for repeatable and predictive developmental and reproductive toxicity assessment in C. elegans
Source: Sci Rep. 2026 Jul 10;16:31643. doi: 10.1038/s41598-026-61532-4 (PMC13649564; doi:10.1038/s41598-026-61532-4)
Supplement: Supplementary file 1 [file 41598_2026_61532_MOESM1_ESM.pdf]

A Robust, High-Content NAM for Repeatable and Predictive Developmental and Reproductive Toxicity Assessment in *C. elegans*

Sudip Mondal[1], Adam Laing[1], Amber Shen[1], Evan Hegarty[1], Abhishri Medewar[1], Sebastian Gomez[1], Gina Carrion[1], Julia Brown[1], Piper Hunt[2], and Adela Ben-Yakar[1,3,4]

[1]vivoVerse, LLC, Austin, TX, 78731, USA
[2]U.S. Food and Drug Administration, Human Foods Program, Office of Chemistry and Toxicology, Laurel, MD, 20708, USA
[3]Department of Mechanical Engineering, The University of Texas at Austin, Austin, TX, 78712, USA
[4]Department of Biomedical Engineering, The University of Texas at Austin, Austin, TX, 78712, USA

Supplementary information

Supplementary Figure 1. Traditional approach to analyze brood size and hatching efficiency.

Supplementary Figure 2. Overview of vivoScreen technology platform and imaging workflow using vivoChip.

Supplementary Figure 3. Screenshot of the vivoImager software during automated imaging of a vivoChip-24x device.

Supplementary Figure 4. An automated image acquisition and analysis pipeline to rapidly screen *C. elegans* models for DART studies**.**

Supplementary Figure 5. Dataset for U-Net-based body segmentation model.

Supplementary Figure 6. Dice score for the predicted worm segmentation.

Supplementary Figure 7. Example of *C. elegans* body segmentation.

Supplementary Figure 8. Screenshot of the vivoAnalyzer software for embryo phenotyping.

Supplementary Figure 9. Workflow of statistical analysis for body and embryo phenotypes.

Supplementary Figure 10. Well averages for all 6 DART parameters from one of the biological replicates.

Supplementary Figure 11. Variation in DART parameters across worms treated with four solvent conditions and five biological replicates.

Supplementary Figure 12. Variability across sixty 3-well combinations from 5 biological and 6 technical replicates.

Supplementary Figure 13. Statistical power estimates for late-stage embryo parameters with different numbers of biological replicates.

Supplementary Figure 14. Range-finding assay with methylmercury.

Supplementary Figure 15. Representative images of methylmercury-treated worms.

Supplementary Figure 16. Concentration-response curve for methylmercury with 10 concentrations.

Supplementary Figure 17. Range-finding assay with propiconazole.

Supplementary Figure 18. Representative images of propiconazole-treated worms.

Supplementary Figure 19. vivoChip-24x automated imaging workflow.

Supplementary Table 1. List of key steps involved in traditional DART assay and vivoDART assay.

Supplementary Table 2. Aggregated average values for six DART parameters from one biological replicate.

Supplementary Table 3. Aggregated average values for six DART parameters from 30 well-level averages.

Supplementary Table 4. Effective concentration values for DART-related parameters for methylmercury-treated worms.

Supplementary Table 5. Effective concentration values for DART-related parameters for methylmercury-treated worms with 10 concentrations.

Supplementary Table 6. Strictly Standardized Mean Difference values for different concentrations of methylmercury.

Supplementary Table 7. Effective concentration values for DART-related parameters for propiconazole-treated worms.

Supplementary Table 8. Predefined assay acceptance criteria for DART experiments.

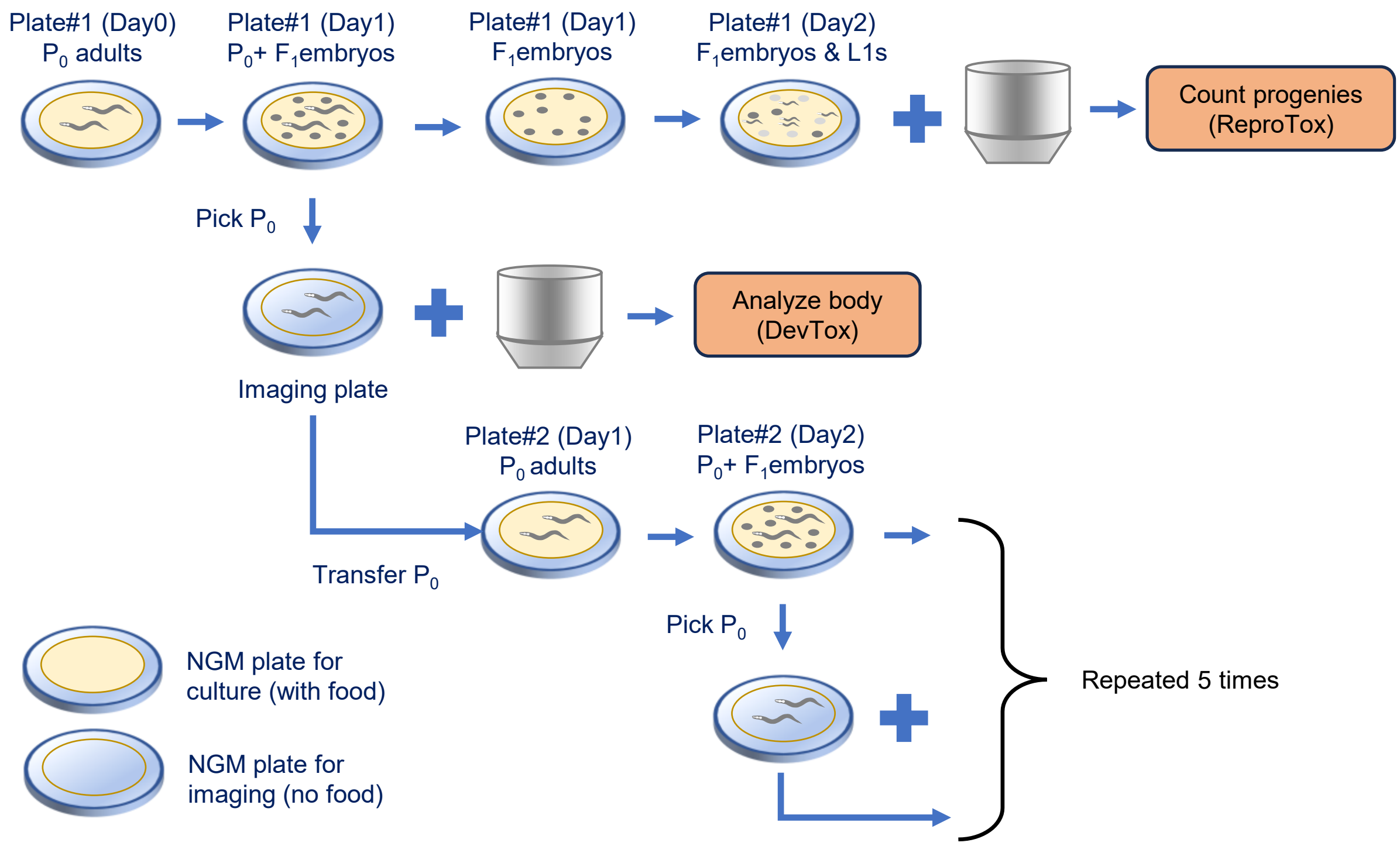

Supplementary Figure 1. Traditional approach to analyze brood size and hatching efficiency. In conventional DART assays, body size, brood size, and hatching efficiency are estimated through repeated daily manual transfer of adult *C. elegans* on NGM plates. Each day, parental adults are removed from the culture plates, washed with buffer, and plated on imaging plates with no bacterial food. The adults are imaged to analyze body sizes for developmental toxicity (DevTox) parameters. After imaging, adults are transferred to a fresh NGM plate with bacterial food for 24 hours. Embryos laid during this period are analyzed the following day to count total number of hatched progenies and unhatched embryos, which are used to assess reproductive toxicity (ReproTox) parameters. This process is repeated for 5-6 days to estimate total brood size and hatching efficiency for each chemical condition. Because the workflow requires repeated handling, multiple plates, and delayed progeny-based readouts, it is labor-intensive and susceptible to sample loss, handling-induced stress, and increased variability, particularly under exposure conditions that cause delayed development, impaired egg laying, or fragile worm bodies.

A

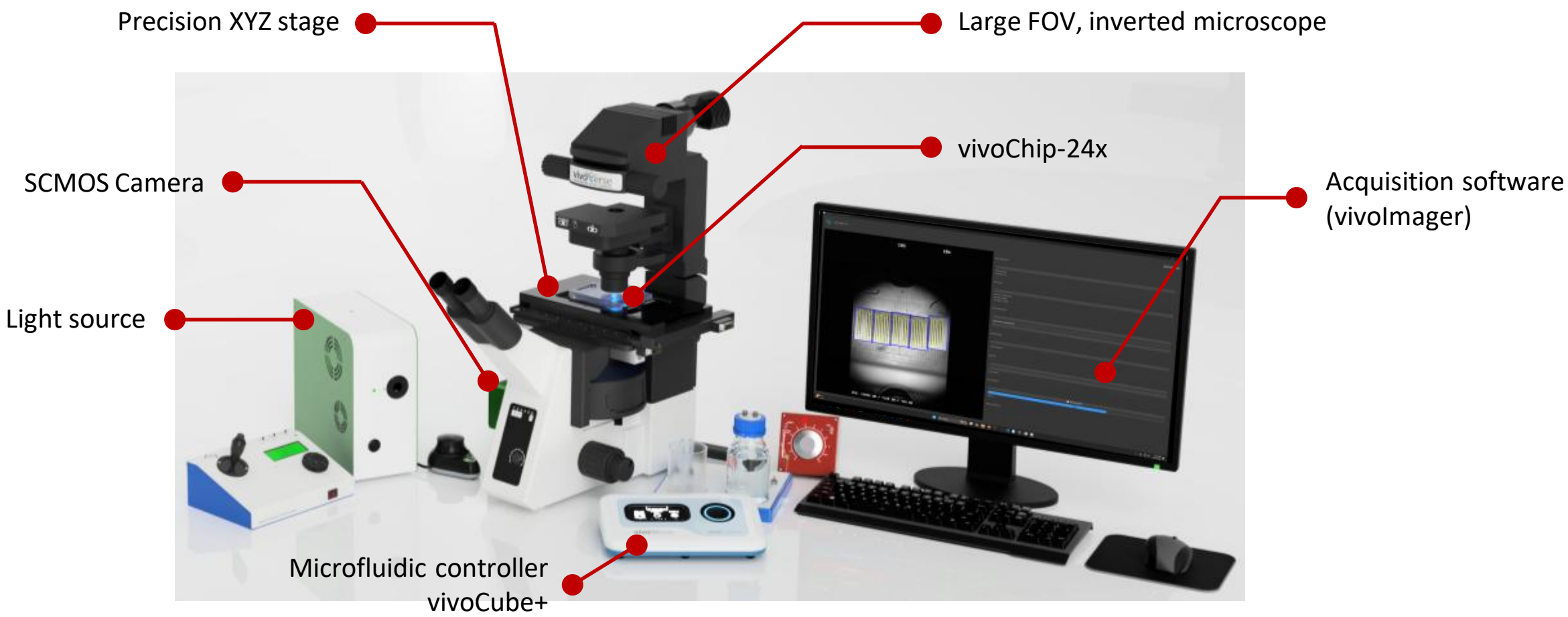

B

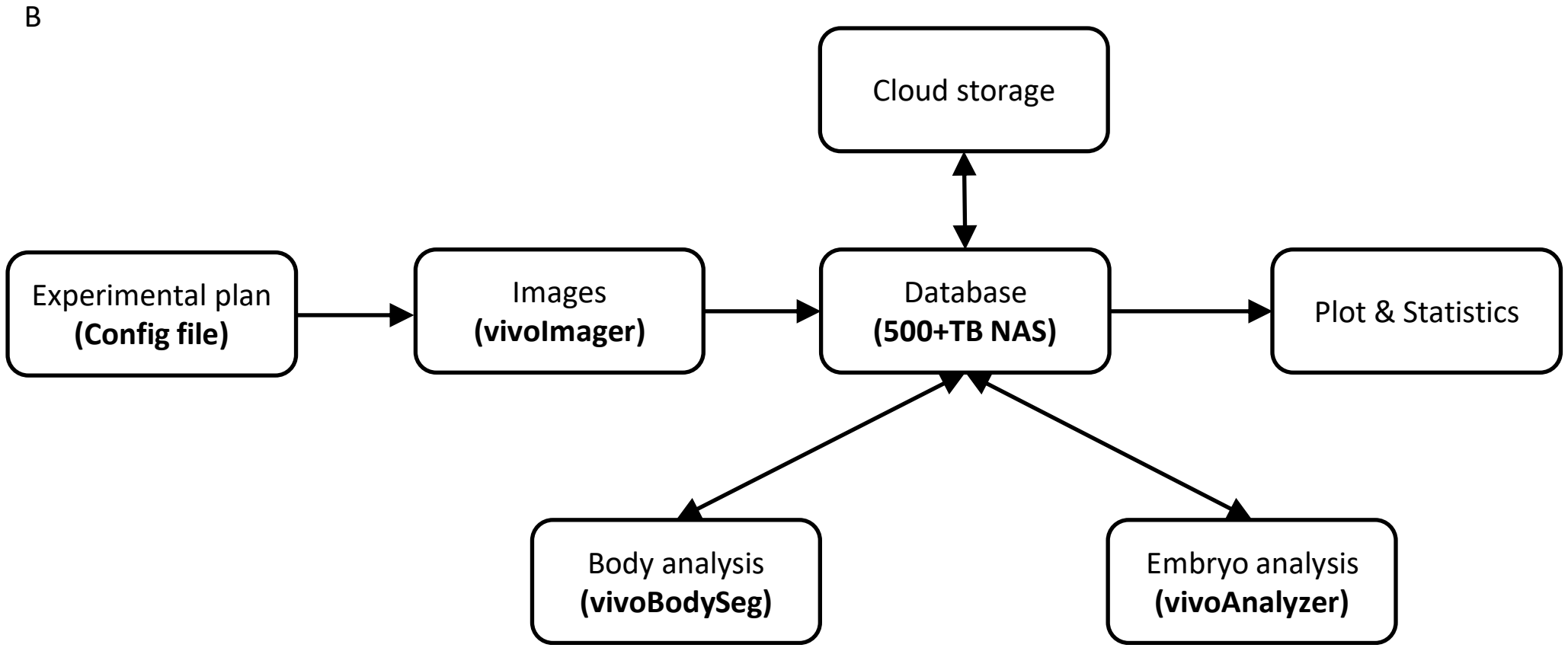

Supplementary Figure 2. Overview of vivoScreen technology platform and imaging workflow using vivoChip. (A) Schematic illustrating the main hardware components of the vivoScreen platform. The system comprises a customized inverted microscope, configured for large field-of-view (FOV) image acquisition using a scientific CMOS camera, high-precision XYZ translational stages, and programable light sources. The hardware is controlled using an in-house image acquisition software (vivoImager), is optimized for brightfield, fluorescence, and timelapse imaging of *C. elegans* immobilized within the vivoChip-24x device. This microfluidic device is operated using a dedicated controller (vivoCube+). **(B)** Each vivoChip-24x imaging experiment starts with preparing a config file, followed by acquiring data using vivoImager with vivoScreen hardware. Data is stored locally using 500+ TB network attached storage (NAS) and on cloud. A centralized, searchable database connects with all software modules and archives config and analysis files. Data is subsequently processed automatically to extract body parameters using a machine-learning (ML) model (vivoBodySeg [1]) and semi-automatically using a graphic user interface (vivoAnalyzer) for embryo phenotype analysis. The resulting data is then used for plots and statistical analyses.

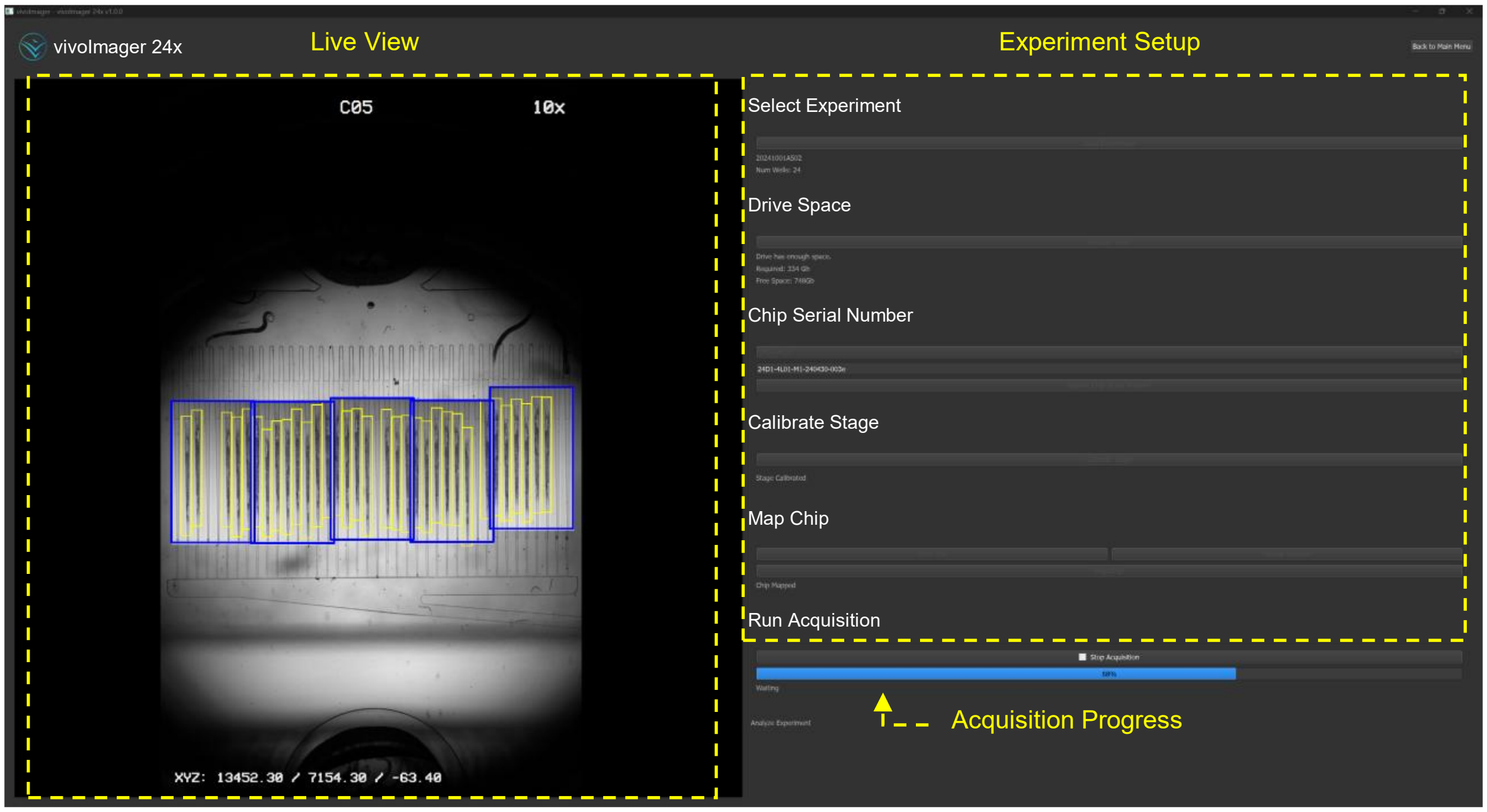

Supplementary Figure 3. Screenshot of the vivoImager software during automated imaging of a vivoChip-24x device. The software interface is organized into three main panels: (1) Experiment Setup Options, (2) Live View, and (3) Acquisition Progress. The Experiment Setup section guides the user through step-by-step process, starting with selection of a specific experiment config file and entry of the chip serial number. The program verifies that sufficient local storage is available before proceeding with the fully automated process for stage calibration, chip mapping, and data acquisition. The Live View panel displays 2× image of the well currently being imaged. Yellow boxes indicate individual bounding boxes identified by the YOLO-based detection model using 2× image. Based on these bounding boxes, the software predicts optimum positions for 5 field of views (FOVs, blue boxes) to capture all 40 channels. The Acquisition Progress panel displays real-time status updates as the software captures brightfield, fluorescence, or timelapse images from all 24 wells with 5 FOVs/well using the vivoChip-24x device.

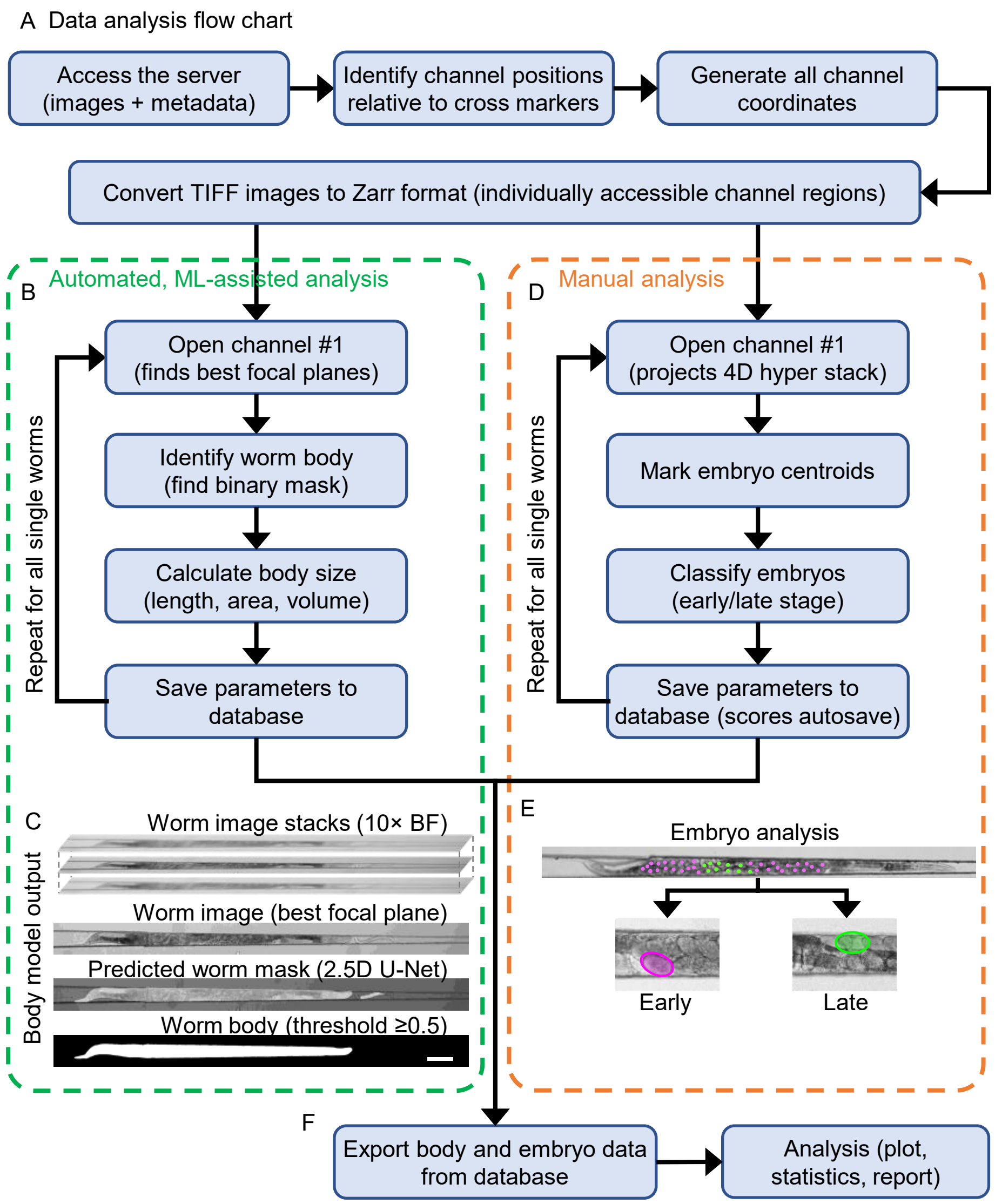

Supplementary Figure 4. An automated image acquisition and analysis pipeline to rapidly screen *C. elegans* models for DART studies. (A) Flowchart showing the image processing pipeline, beginning with accessing raw imaging data, identifying channel positions relative to fiduciary cross markers, and registering boundary coordinates for individual channels. The associated metadata are stored on the server. Raw TIFF stacks are converted to the Zarr format, which allows data-efficient access to image subsets corresponding to an individual channel withing the full FOV (8 channels per FOV). This format allows parallel read and write operations across multiple threads or processes, facilitating distributed computing frameworks necessary for high-speed analysis. **(B)** Overview of the automated, ML-assisted body segmentation process. The model selects the best 5 focal planes, predicts a body mask, generates a binary segmentation of the worm body outline, and computes body parameters including body length, area, and volume of the worm. **(C)** Representative images of a worm within a single channel showing the steps of automated ML-assisted body segmentation (finding the best focus plane from the z-stack, predicting body pixels using the trained model, and generating a binary mask by thresholding pixels with a probability >0.5 of belonging to the worm body). Scale bar is 100 µm. **(D)** Overview of the manual

embryo phenotyping workflow, in which trained human scorers label and categorize all embryos within the germline of each worm. **(E)** Representative images of an individual worm showing the embryo scoring (marking the centroid) and classification (early- or late-stage embryo) by human users. **(F)** Scoring and classification data from each experiment is stored in a database and can be exported for plotting and statistical analysis.

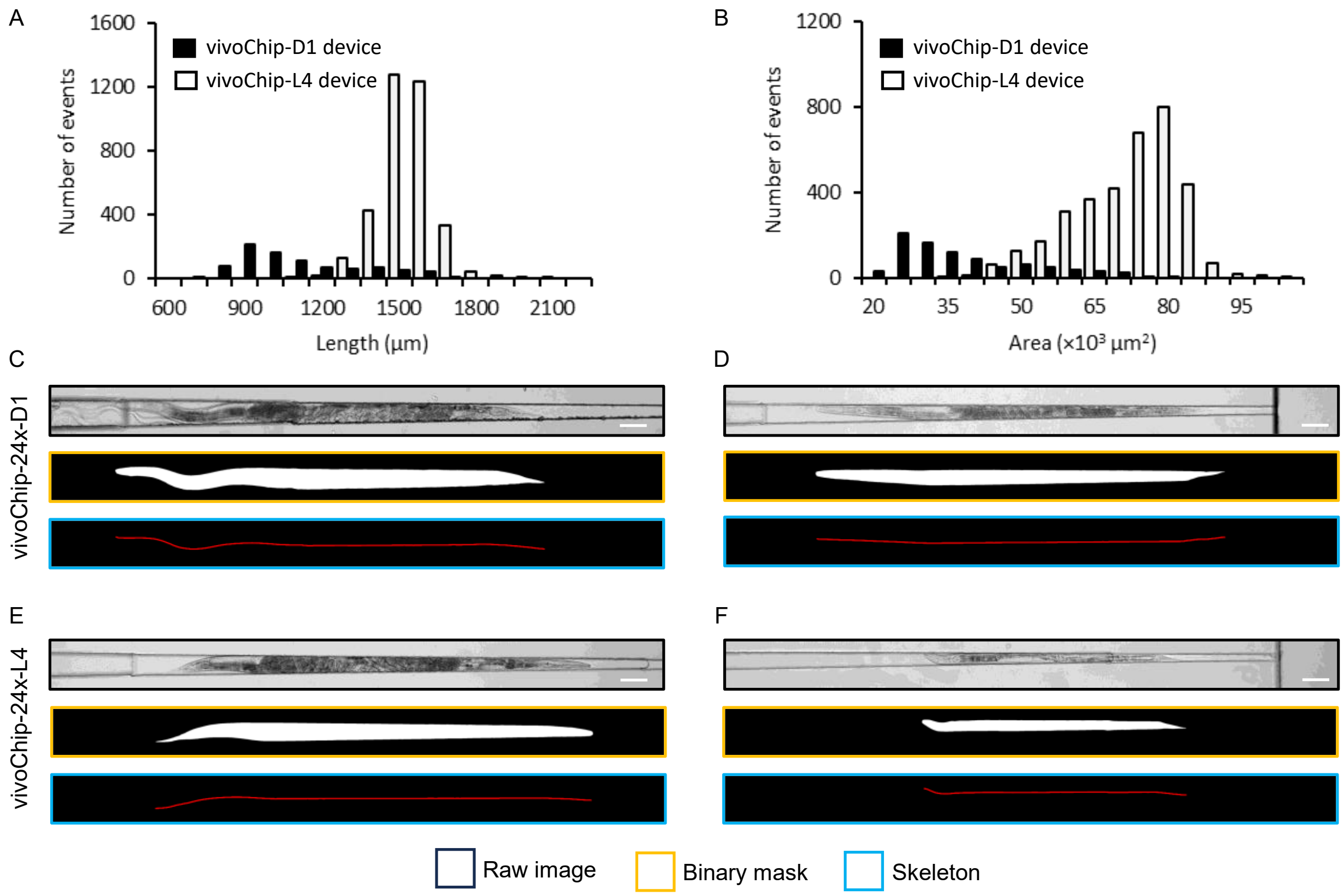

Supplementary Figure 5. Dataset for U-Net-based body segmentation model. The training dataset consists of n = 4,360 channels, manually classified as full and segmented for the worm bodies. **(A-B)** Histogram plot of body length (A) and area (B) quantified from the manually segmented body masks. **(C-F)** Example images of worms immobilized in the vivoChip-24x-D1 (C-D) and vivoChip-24x-L4 (E-F) devices.

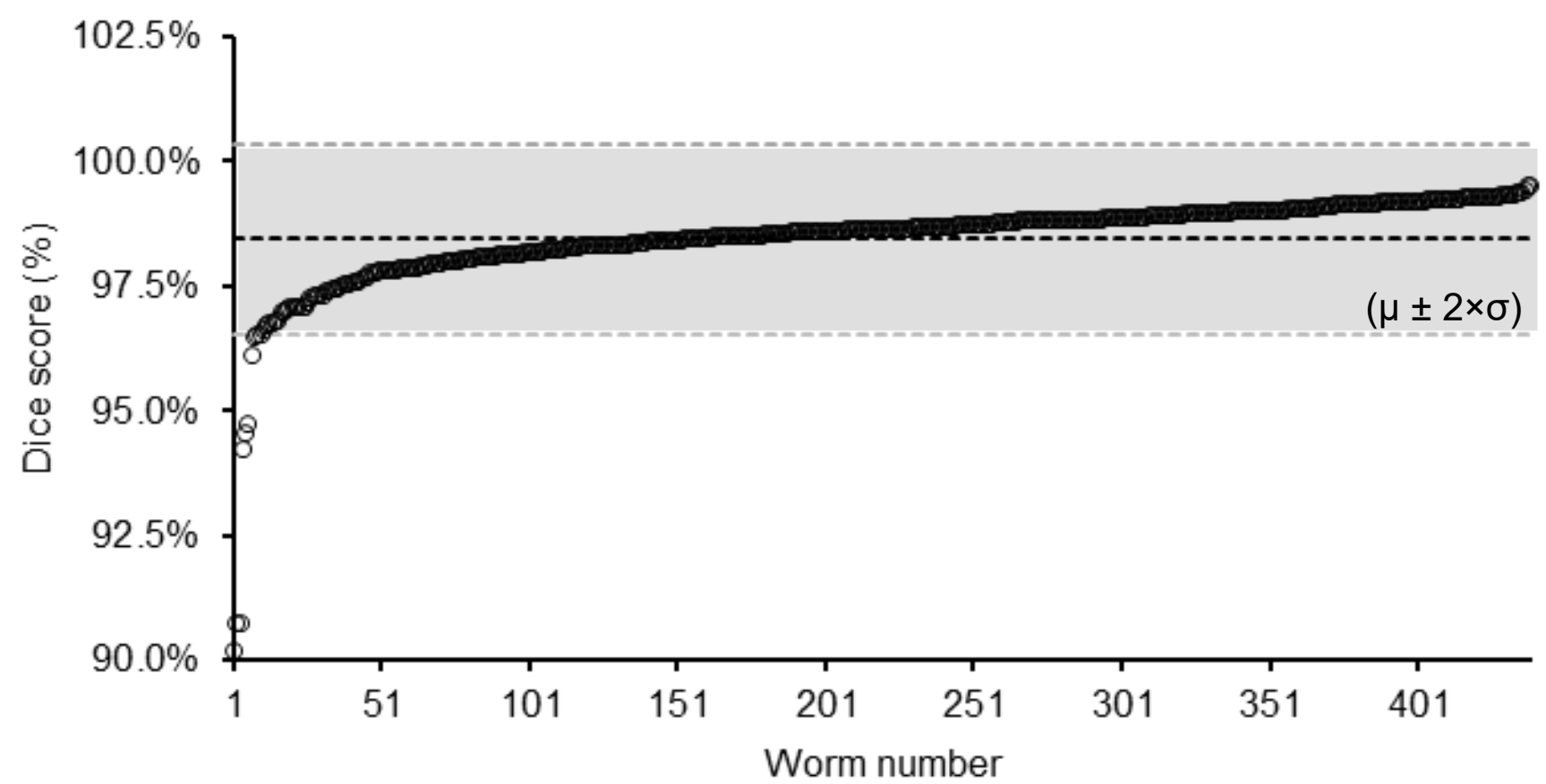

Supplementary Figure 6. Dice score for the predicted worm segmentation. The Dice score for all 439 test channels with full worms are arranged with worm numbers representing low to high Dice scores. The grey area represents the mean ± 2×standard deviation (μ ± 2σ) values for the Dice score. Only 8 channels were below 96.5% (μ - 2σ) dice score.

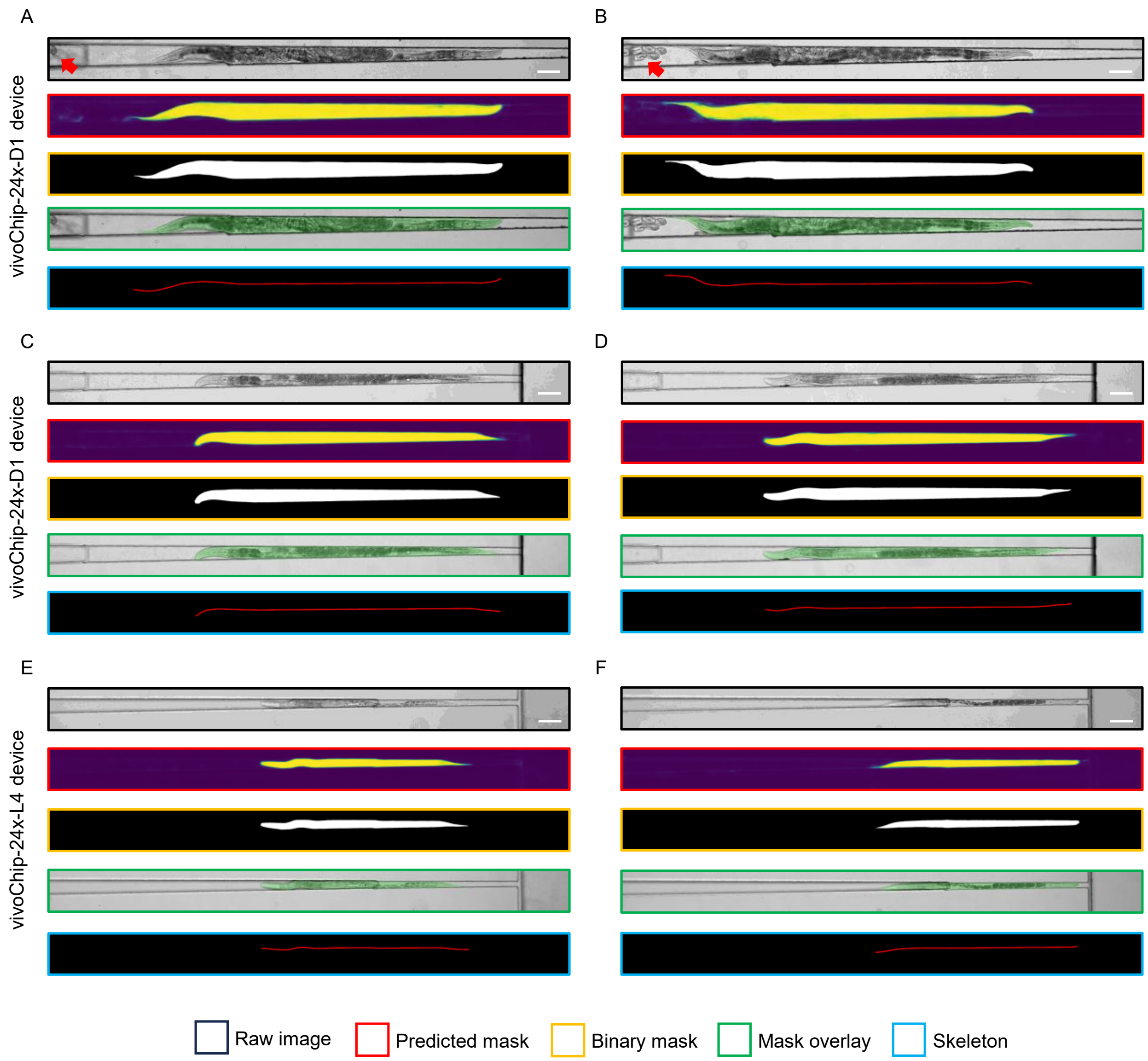

Supplementary Figure 7. Example of *C. elegans* body segmentation. (A-D) Example worm imaged using vivoChip-24-D1 device. **(E-F)** Example worm imaged using vivoChip-24-L4 device. The image shows the raw image, predicted mask, binary mask, overlaid mask, and skeleton. The skeleton is used for body length estimate (red line), region within the binary mask is used to estimate body area, and channel height is used for volume calculation. The red arrow indicates an unhatched egg inside the channel, which is ignored since it is outside the large worm body mask. The scale bar = 200 µm.

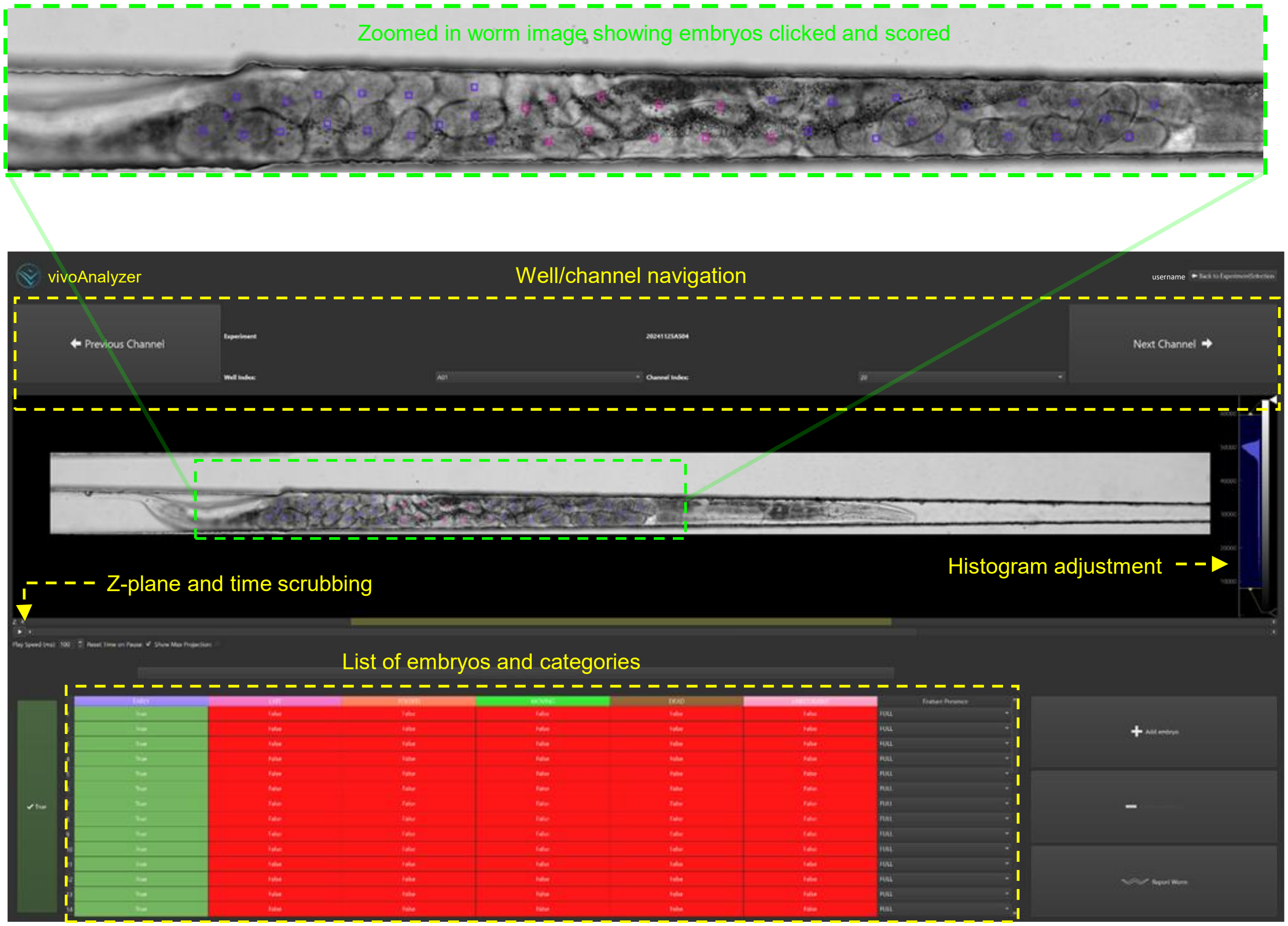

Supplementary Figure 8. Screenshot of the vivoAnalyzer software for embryo phenotyping. The vivoAnalyzer software is used to load individual imaging channels with worms for manual embryo scoring. Users can adjust the image histogram, scroll through the full z-stack, and zoom into specific regions in the image to facilitate accurate scoring and classification. When an embryo is identified, the user scores the embryo by clicking on the center of the embryo and assigns it to one of the 2 classifications: early-stage embryo or late-stage embryo. After scoring and classifying all embryos within a channel, the user proceeds to the next channel. All user inputs, including click coordinates (vertices), assigned classes (early, late, or none), and user metadata are stored in the centralized database. The vivoAnalyzer operates in two modes; (1) Normal Assignment Mode, in which the experiment name, well name, and channel numbers are visible to the user and (2) Blinded Assignment Mode, in which no identifying information about the channel image is displayed.

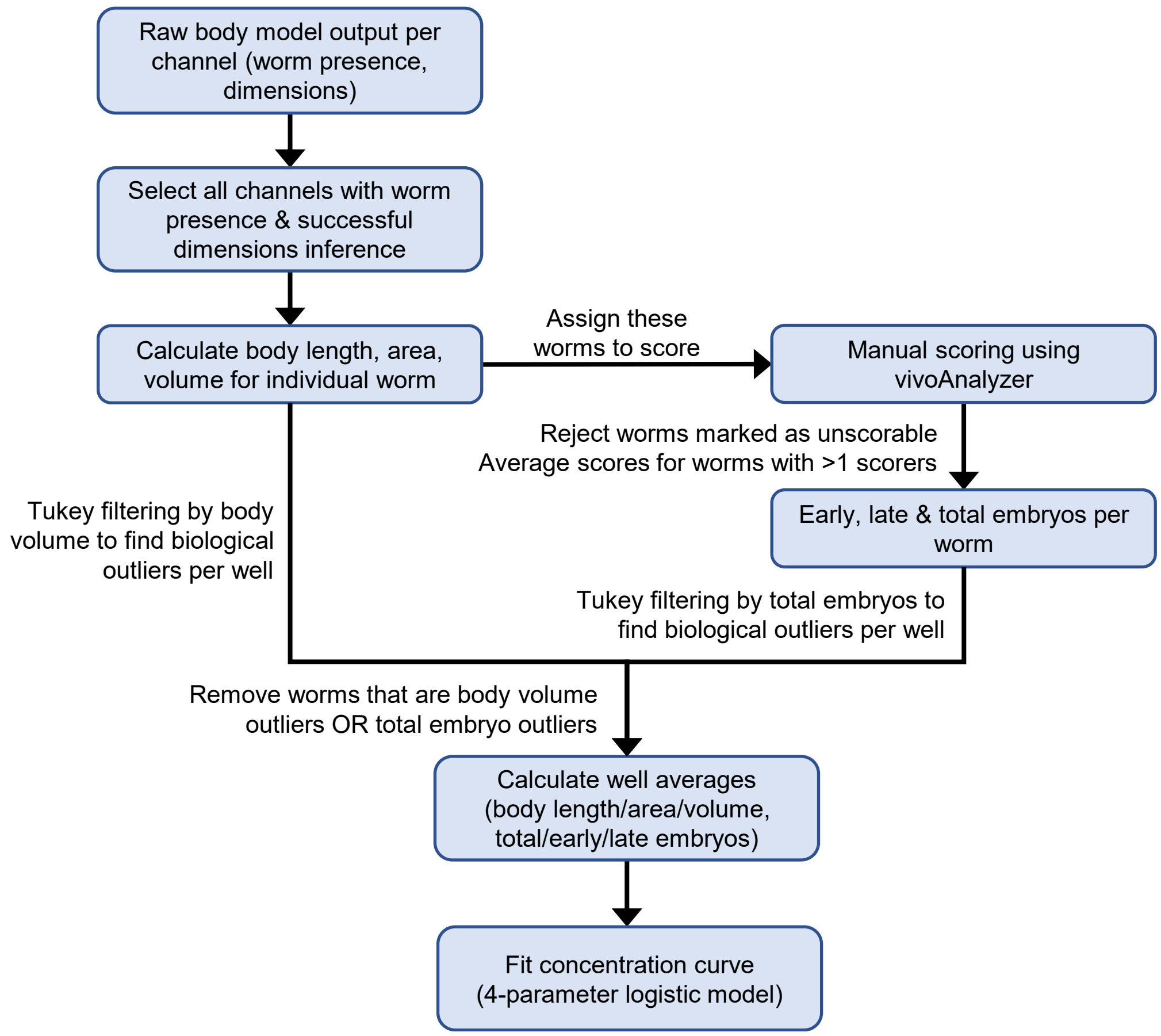

Supplementary Figure 9. Workflow of statistical analysis for body and embryo phenotypes. Raw outputs from body segmentation model, including the worm presence (full, partial, or none) and the predicted segmentation masks (vertices of the polygon, generated only for channels classified as "full") are stored in SQLite (SQLT) files in our NAS data storage system. The developmental parameters are subsequently extracted from the segmentation masks, as described in the Methods section. The channels with worms are scored manually to detect all in utero embryos and classified as early- or late-stage embryos. The manual scores are stored in the database. For each well, we identify the outliers for body volume and total embryos using Tukey filtering. The remaining channels are used to estimate well averages for 3 developmental (length, area, and volume) and 3 reproductive (total, early, and late embryos) parameters. The well averages are used to create concentration curves and fit 4-parameter logistic model to estimate effective concentrations.

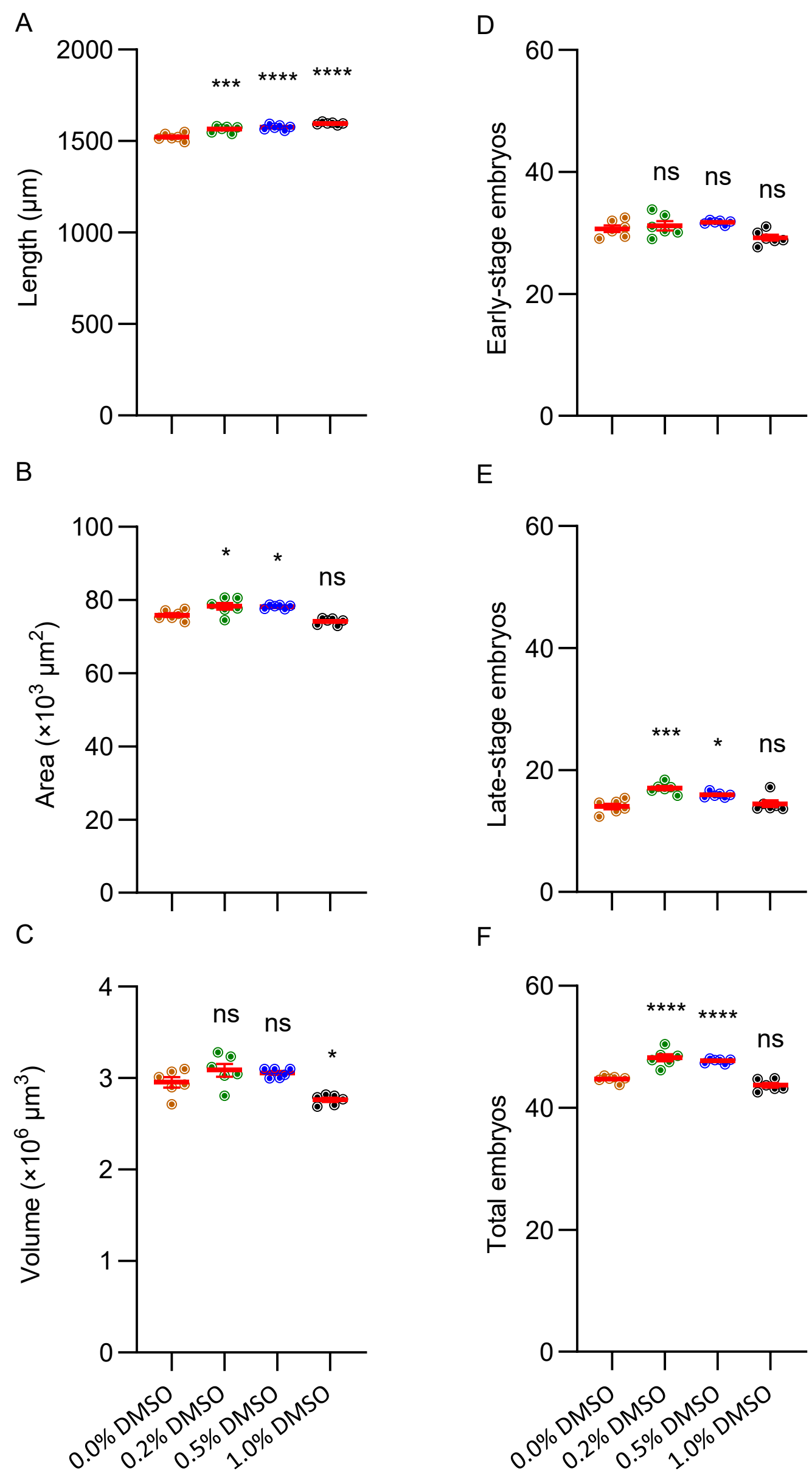

Supplementary Figure 10. Well averages for all 6 DART parameters from one of the biological replicates. Scatter plots showing well averages from all 24 wells for the biological replicates, presented in Fig. 3 in the manuscript. **(A-C)** Scatter plot for body length (A), area (B), and volume (C). **(D-F)** Scatter plot for number of early-stage embryos (D), number of late-stage embryos (E), and number of total embryos (F) per worm. The red lines indicate mean ± SEM. One-way ANNOVA was used to compare the mean values (n = 6 technical replicates) for 0.2%, 0.5%, and 1.0% conditions with respect to 0.0% DMSO wells. The p-values are indicated as ns (p-value ≥ 0.05), * (p-value ≥ 0.05), ** (p-value ≤ 0.01), *** (p-value ≤ 0.001), and **** (p-value ≤ 0.0001).

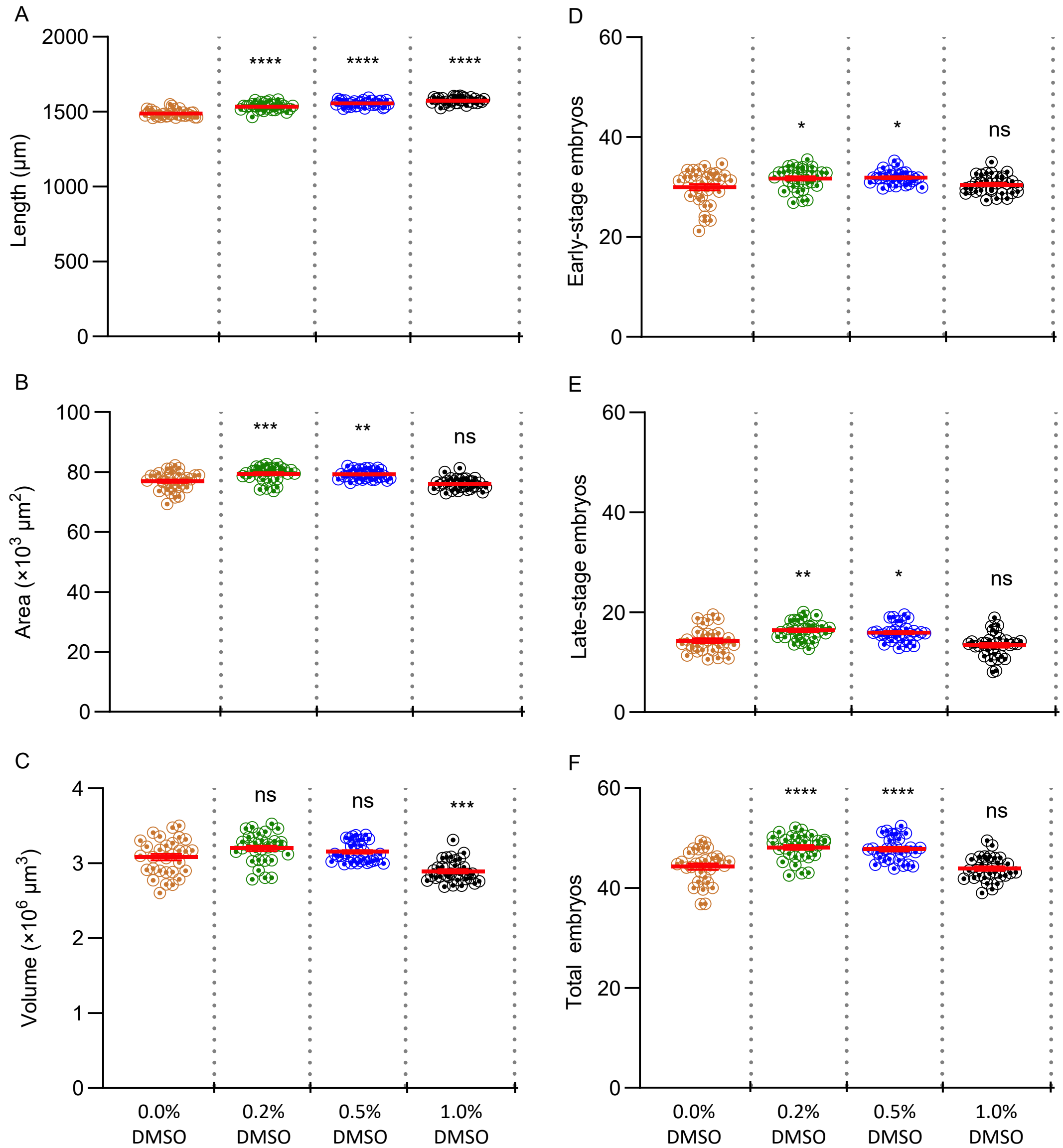

Supplementary Figure 11. Variation in DART parameters across worms treated with four solvent conditions and five biological replicates. Scatter plots showing all 30 well averages (5 biological replicates × 6 technical replicates) for 6 DART-related parameters under four DMSO conditions (0% = Brown, 0.2% = Green, 0.5% = Blue, and 1% = Black). **(A-C)** Scatter plot for body length (A), area (B), and volume (C). **(D_F)** Scatter plot for number of early-stage embryos (D), number of late-stage embryos (E), and number of total embryos (F) per worm. The red lines indicate mean ± SEM. One-way ANNOVA was used to compare the mean values (n = 30 wells each) for 0.2%, 0.5%, and 1.0% conditions with respect to 0.0% DMSO wells. The p-values are indicated as ns (p-value ≥ 0.05), * (p-value ≥ 0.05), ** (p-value ≤ 0.01), *** (p-value ≤ 0.001), and **** (p-value ≤ 0.0001).

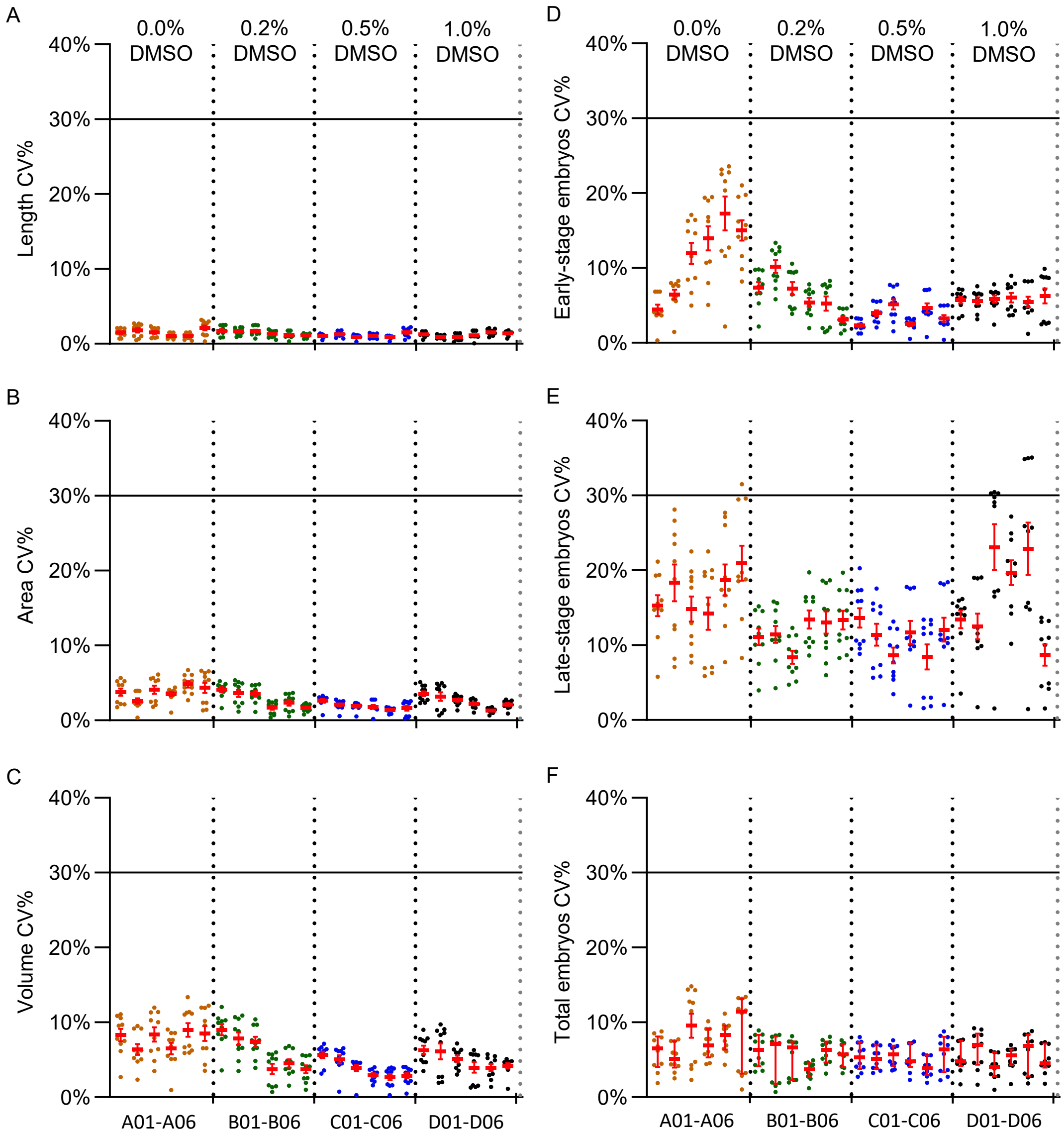

Supplementary Figure 12. Variability across sixty 3-well combinations from 5 biological and 6 technical replicates. (A-F) CV% values for body length (A), body area (B), body volume (C), number of early-stage embryos (D), number of late-stage embryos (E), and number of total embryos (F). Each point represents the CV% derived from a unique combination of 3 corresponding wells across different experiments. The red horizontal line represents the mean ± SEM CV% values across all combinations for each DMSO concentration. The horizontal solid black line represents the 30% CV threshold. The vertical dashed lines separate the four DMSO conditions: A01-A06, B01-B06, C01-C06, and D01-D06.

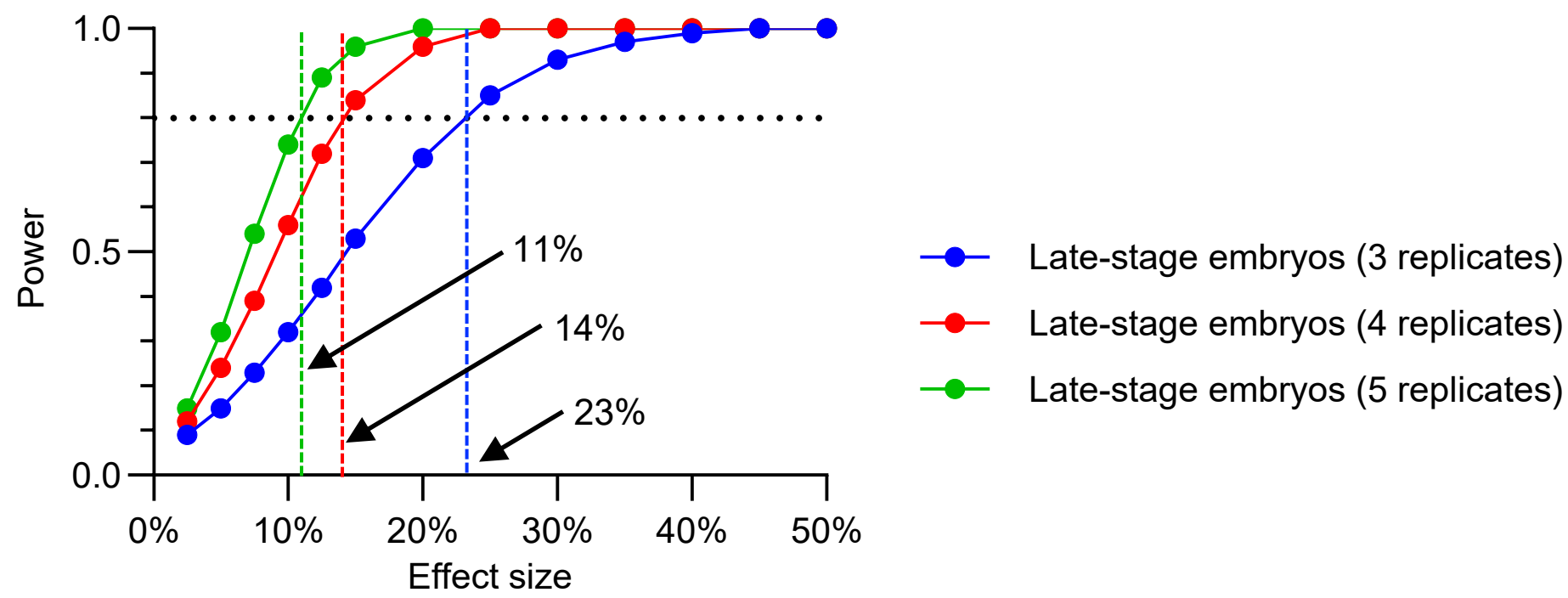

Supplementary Figure 13. Statistical power estimates for late-stage embryo parameters with different numbers of biological replicates. Line and dot plot for the statistical power in number of late-stage embryos using aggregated data from 3 (blue), 4 (red), and 5 biological (green) replicates. The power was estimated to be 2.5 to 50% effect size. The horizontal dotted line represents 80% power. The vertical dotted lines represent the minimum effect sizes for 3, 4, and 5 biological replicates.

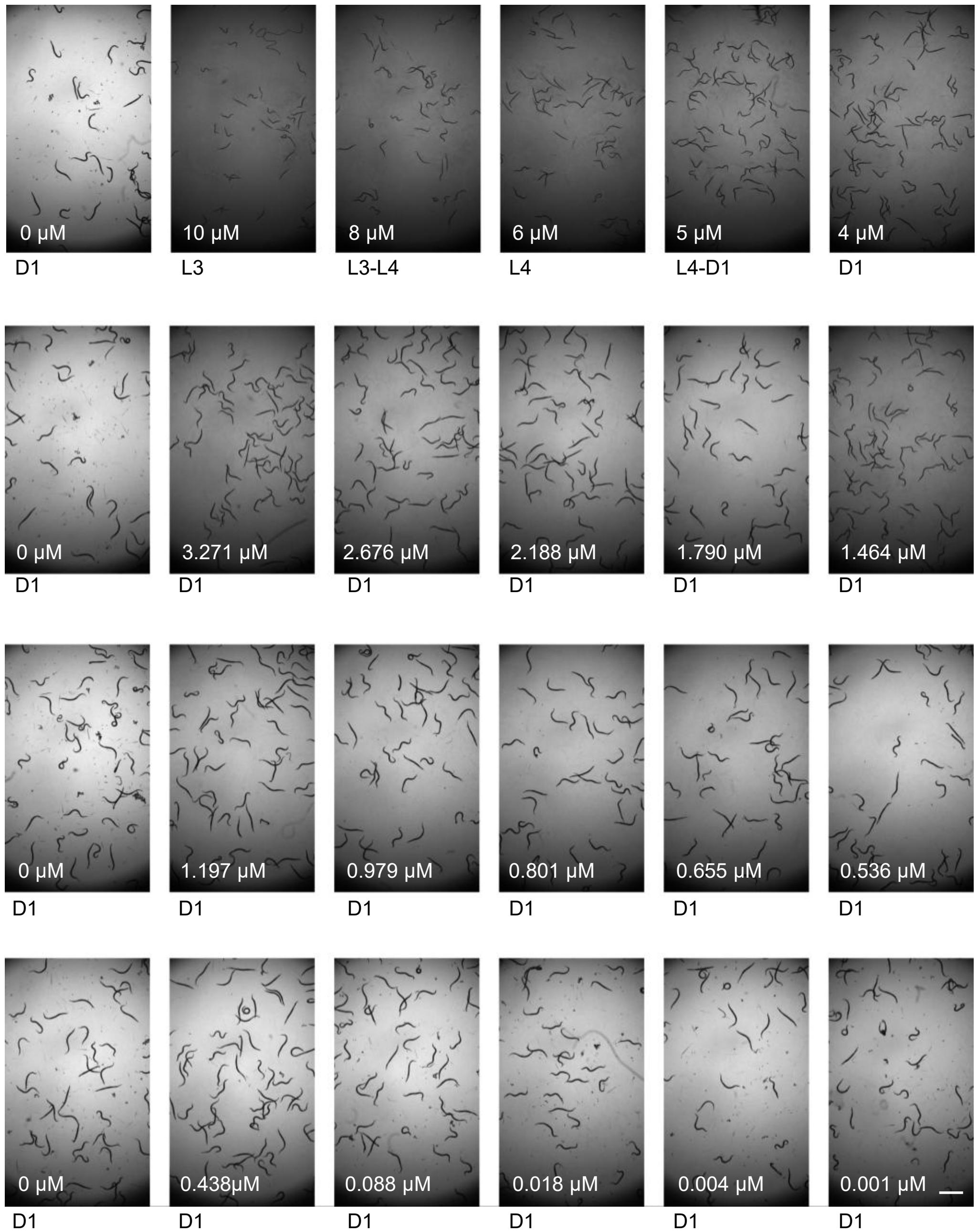

Supplementary Figure 14. Range-finding assay with methylmercury. Worms were treated to increasing concentrations of methylmercury as indicated in the image. The worm developmental stages were manually assessed and the average stage for each condition is shown below the corresponding image. Wells containing worms below the L4 stage were not loaded into the vivoChip-24x device (adult device or L4 device) for high-resolution imaging. Adult worms from 0 up to 4 µM conditions were loaded in the vivoChip-24x (adult device), whereas worms from higher than 4 µM condition were loaded into the vivoChip-24x (L4 device). The scale bar is 200 µm.

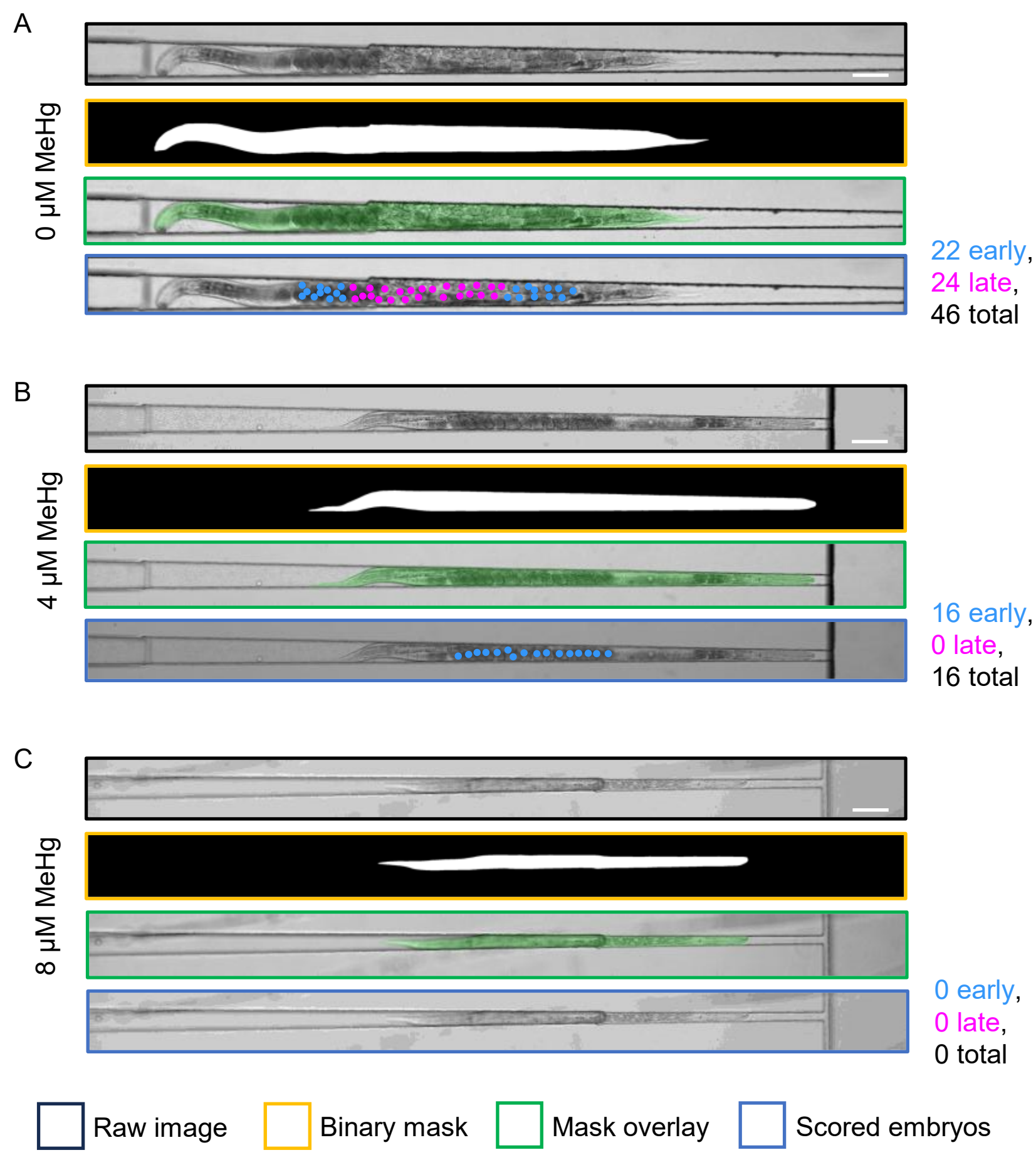

Supplementary Figure 15. Representative images of methylmercury-treated worms. (A-C) Statistically representative example worms selected based on body volume for 0 µM, 4 µM and 8 µM methylmercury (MeHg) treatment conditions. For each channel, the raw image is shown along with the corresponding binary mask predicted by the ML model inference. The third image shows the green-colored overlay of the mask on the worm body. The manually annotated embryo locations and classifications are also shown (blue dots = early-stage embryos and magenta dots = late-stage embryos). Adult worms from the 0 µM and 4 µM conditions were loaded in the vivoChip-24x (adult device), whereas L4-stage worms from the 8 µM condition were loaded into the vivoChip-24x (L4 device). Scale bar is 100 µM.

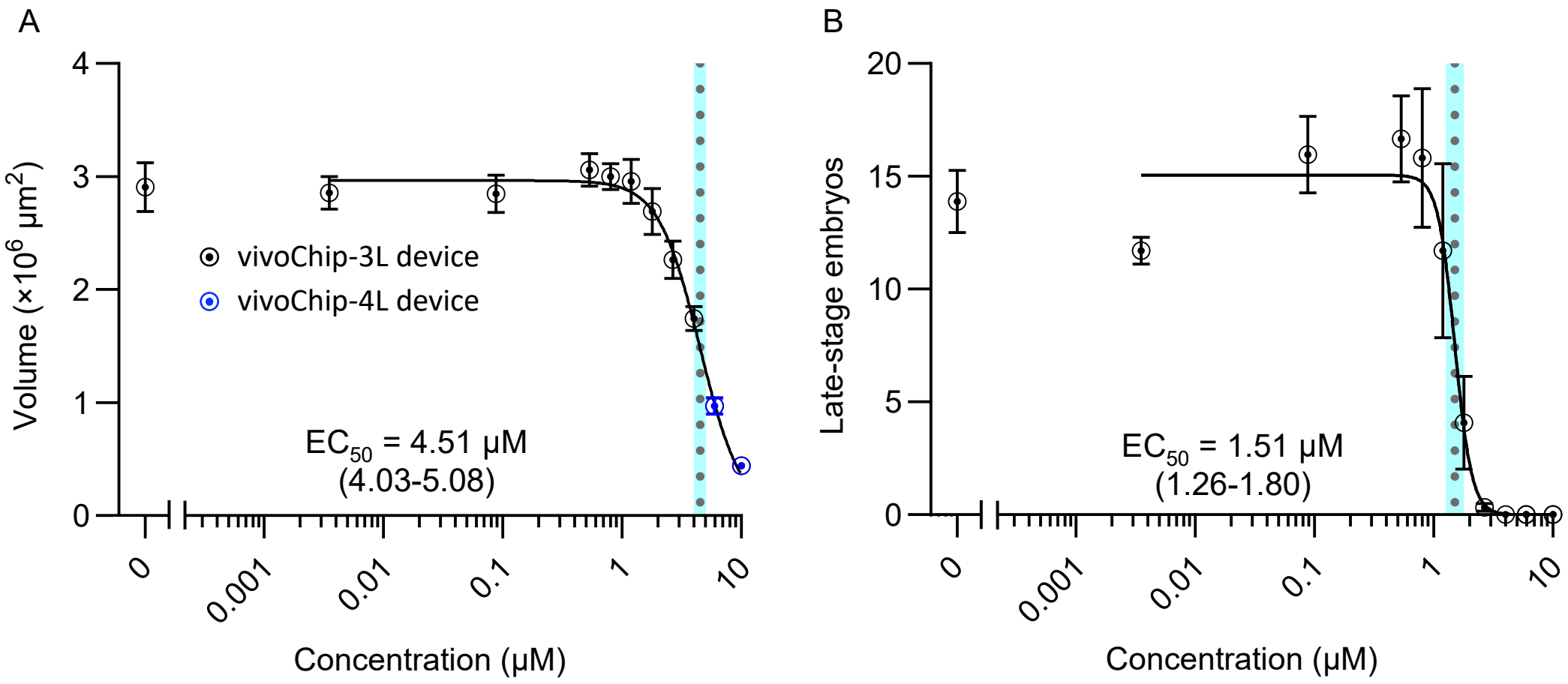

Supplementary Figure 16. Concentration-response curve for methylmercury with 10 concentrations. **(A-B)** The effects of methylmercury on body volume (A) and number of late-stage embryos (B) per worm. We skipped every other data presented in Figure 7 and were used to create a 10-point concentration-response curve for methylmercury. Data from 3 biological replicates were performed using either D1 (black circle) or L4 (blue circles) chips. The combined well average values from both datasets were fitted with a 4-parameter logistic curve (solid black lines). The EC_{50} estimates are indicated with dotted lines with corresponding 95% confidence interval bands shaded in light-blue color.

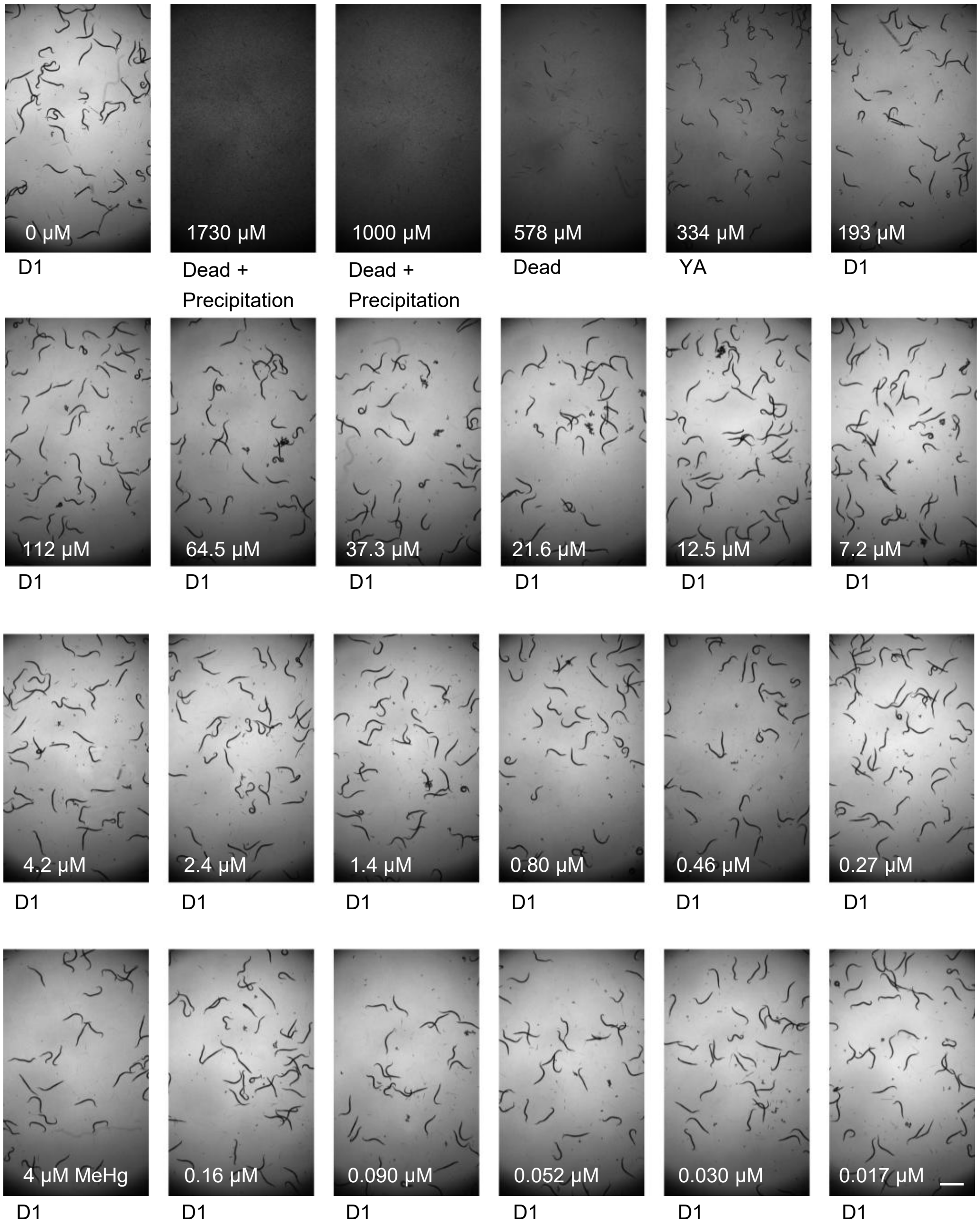

Supplementary Figure 17. Range-finding assay with propiconazole. Worms were treated to increasing concentrations of propiconazole as indicated in the image. The worm developmental stages were manually assessed, and the average stage for each condition is labelled below the corresponding image. Dead worms and any observed precipitation of propiconazole in the culture medium are also indicated. Wells containing worms below the L4 stage were not loaded into the vivoChip-24x device (adult device or L4 device) for high-resolution imaging. Adult worms from 0 up to 193 µM conditions were loaded in the vivoChip-24x (adult device), whereas worms from 334 µM was loaded into the vivoChip-24x (L4 device). The scale bar is 200 µm.

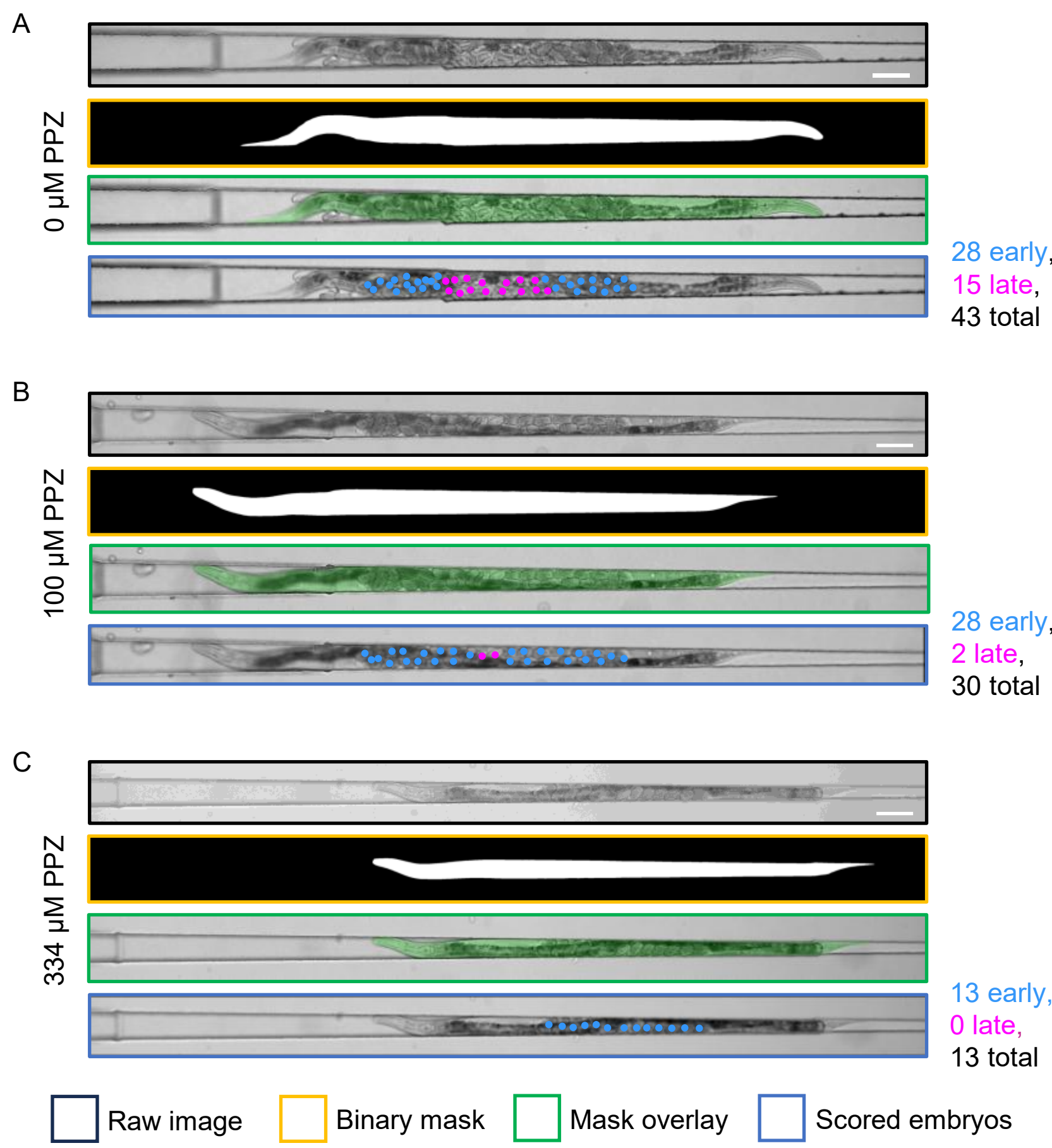

Supplementary Figure 18. Representative images of propiconazole-treated worms. (A-C) Statistically representative example worms selected based on body volume for 0 µM, 100 µM and 334 µM propiconazole (PPZ) treatment conditions. For each channel, the raw images are shown along with the binary mask predicted by the ML model inference. The third image shows the green-colored overlay of the mask on the worm body. Manually annotated embryo locations and classifications are also shown (blue dots = early-stage embryos and magenta dots = late-stage embryos). Adult worms from the 0 µM and 100 µM conditions were loaded into the vivoChip-24x (4L device), whereas worms from the 334 µM condition were loaded into the vivoChip-24x (D1 device), as approximately 34% of this worm population was below the minimum size that could be reliably trapped in the vivoChip-24x (L4 device). Scale bar is 100 µM.

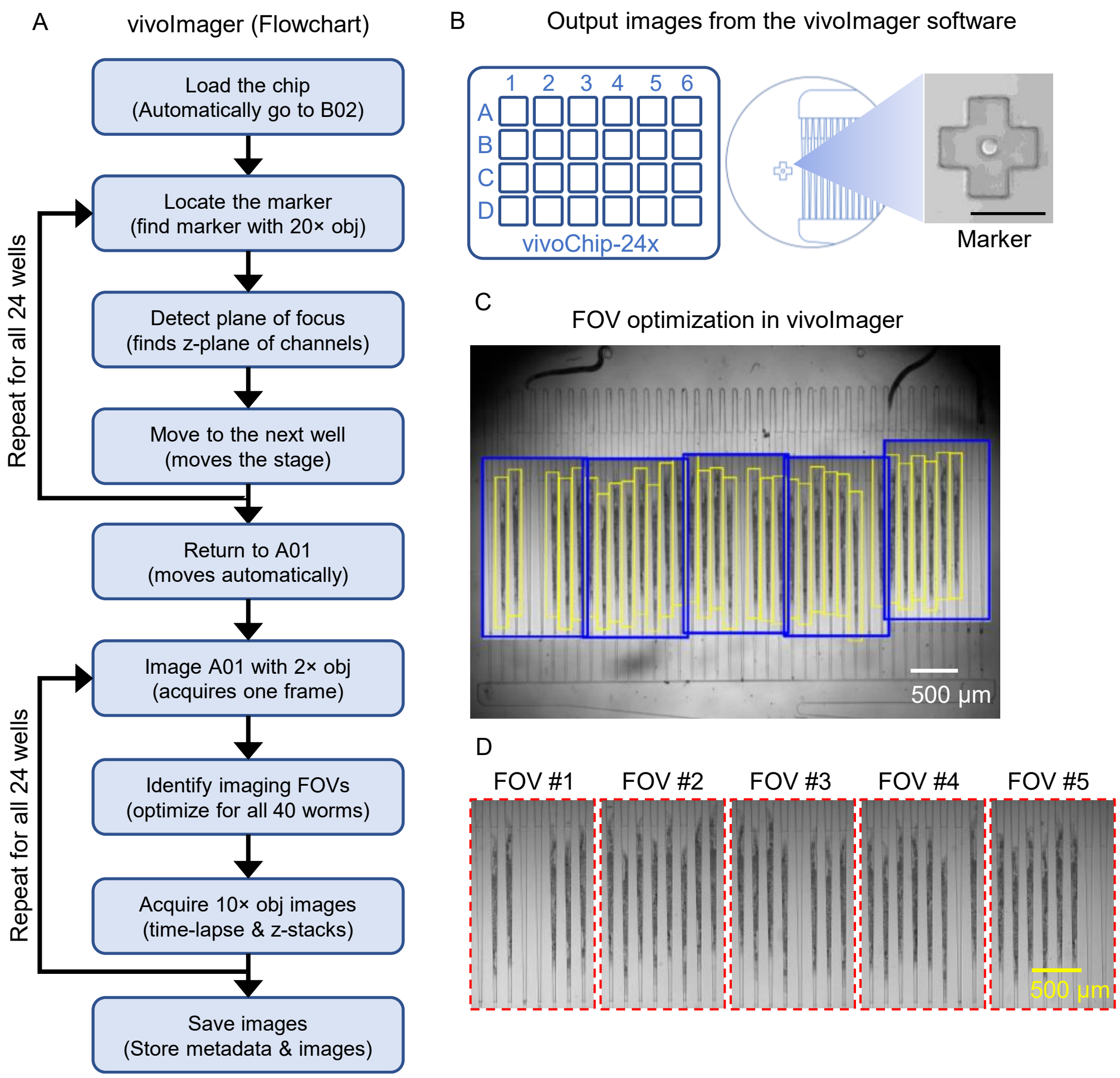

Supplementary Figure 19. vivoChip-24x automated imaging workflow. (A) Flowchart illustrating the automated imaging workflow. **(B)** Fiduciary cross markers positioned adjacent to each device are used to find the best focal plane prior to image acquisition. **(C)** Bounding boxes (yellow) corresponding to individual worms trapped within the microfluidic channels are identified using a worm-detection ML model applied to 2× objective (2× obj) images. Based on the detected worm positions, the software computes 5 FOVs that maximize centering of each group of 8 adjacent worms in xy within the imaging bounds of a 10× objective. The calculated FOV positions are overlaid as blue boxes on the 2× image. **(D)** The 5 FOVs capture all 40 channels at the optimal xy locations, enabling whole-body imaging for the majority of the *C. elegans* within the device.

Supplementary Table 1: List of key steps involved in traditional DART assay and vivoDART assay.

Steps	Traditional approach / time needed	vivoDART assay / time needed	Comments
Preparing NGM plates[1]	1050 plates / 52.5 hours	6 plates / 0.3 hours	Consumable cost is 175× higher in conventional approach than vivoDART assay
Preparing exposure plates	36 NGM plates / ~6 hours	3 × 24-well plates / 1.5 hours	Exposure is non-uniform on NGM plates and not suitable for volatile chemicals
Transferring adult worms manually[2]	8,664 adults / 58 hours	24 adults / 0.1 hours	vivoDART requires 8 adults for synchronization for each of the 3 biological replicates.
Measure body	1044 NGM / 75 hours	1440 adults / 0.13 hours	AI-accelerated analysis takes <5 minutes to analyze ~1,000 worms.
Counting progenies	1044 NGM plates / 75 hours[3]	Count in utero embryos / 72 hours[4] (0.13 hours with AI[5])	Scoring on NGM plate is difficult due to poor image contrast on bacteria lawns.
Total time	266.5 hours	74.03 hours[4] (2.16 hours with AI[5])	vivoDART is 3.6× and 123× faster with manual and machine learning-based analysis.

[1]Assum 10 adults per NGM plate is used and it takes ~2 hours to prepare 40 NGM plates.
[2]Total number of 1,440 *C. elegans* are analyzed for one chemical with 10 concentrations, 2 assay controls, 40 worms per condition, and 3 biological replicates. It is estimated that manual handling of 10 worms takes 2 minutes.
[3]It is estimated that both body (manual imaging and automated analysis) progeny analysis (manual imaging and analysis) takes 5 minutes per plate per assay.
[4]Manual analysis of in utero embryos takes ~3 minutes per worm for vivoDART assay.
[5]With AI/machine learning-based approach we can analyze one chip in less than 5 minutes.

Supplementary Table 2. Aggregated average values for six DART parameters from one biological replicate. For each DMSO condition, aggregated average values were calculated from the well-level averages from 6 technical replicates. One-way ANNOVA was used to compare the mean values (n = 6 wells) for 0.2%, 0.5%, and 1.0% conditions with respect to 0.0% DMSO wells.

**Parameter**	**0% DMSO**	**0.2% DMSO** (*p*-value)	**0.5% DMSO** (*p*-value)	**1.0% DMSO** (*p*-value)
Body length (µm)	1522	1565 (0.0005)	1575 (<0.0001)	1596 (<0.0001)
Body area (μm^2)	75891	78289 (0.0414)	78216 (0.0497)	74150 (0.1878)
Body volume (μm^3)	2954407	3085628 (0.2381)	3056318 (0.4459)	2763097 (0.0451)
Early-stage embryos	30.7	31.2 (0.9161)	31.8 (0.4985)	29.2 (0.2287)
Late-stage embryos	14.0	17.0 (0.0003)	15.9 (0.0190)	14.5 (0.8577)
Total embryos	44.7	48.2 (<0.0001)	47.7 (<0.0001)	43.7 (0.2354)

Supplementary Table 3. Aggregated average values for six DART parameters from 30 well-level averages. For each DMSO condition, we used thirty well-level averages from 5 biological and 6 technical replicates. One-way ANNOVA was used to compare the mean values (n = 30 wells) for 0.2%, 0.5%, and 1.0% conditions with respect to 0.0% DMSO wells.

**Parameter**	**0% DMSO**	**0.2% DMSO** (*p*-value)	**0.5% DMSO** (*p*-value)	**1.0% DMSO** (*p*-value)
Body length (µm)	1488	1535 (<0.0001)	1556 (<0.0001)	1576 (<0.0001)
Body area (μm^2)	76957	79440 (0.0005)	79187 (0.0022)	76102 (0.503)
Body volume (μm^3)	3199407	3152630 (0.0785)	2892833 (0.4719)	3152630 (0.0007)
Early-stage embryos	30.0	31.7 (0.0253)	31.9 (0.0114)	30.5 (0.8140)
Late-stage embryos	14.3	16.4 (0.0021)	15.9 (0.0288)	13.4 (0.3357)
Total embryos	44.3	48.1 (<0.0001)	47.8 (<0.0001)	43.9 (0.9373)

Supplementary Table 4. Effective concentration values for DART-related parameters for methylmercury-treated worms. The EC_{50}, EC_{25}, and EC_{10} and 95% confidence interval (95% CI) are calculated for all six parameters. The EC_{10} values for data after normalization with respect to the controls are also shown.

**Parameters**	**EC_{50} (95% CI)**	**EC_{25} (95% CI)**	**EC_{10} (95% CI)**	**Normalized data EC_{10} (95% CI)**
Length	8.22 µM (7.83-8.69)	4.90 µM (4.65-5.15)	2.92 µM (2.65-3.18)	3.62 µM (3.33-3.92)
Area	5.33 µM (4.98-5.72)	3.21 µM (2.91-3.52)	1.93 µM (1.63-2.24)	2.11 µM (1.95-2.28)
Volume	4.61 µM (4.28-4.99)	2.93 µM (2.62-3.26)	1.86 µM (1.56-2.20)	1.97 µM (1.79-2.16)
Early-stage embryos	3.90 µM (3.66-4.16)	3.26 µM (2.98-3.54)	2.72 µM (2.38-3.08)	3.01 µM (2.62-3.42)
Late-stage embryos	1.49 µM (1.33-1.67)	1.25 µM (1.07-1.42)	1.04 µM (0.82-1.26)	1.08 µM (0.92-1.24)
Total embryos	3.14 µM (2.90-3.38)	2.32 µM (2.07-2.58)	1.72 µM (1.44-2.01)	1.94 µM (1.71-2.19)

Supplementary Table 5. Effective concentration values for DART-related parameters for methylmercury-treated worms with 10 concentrations. The EC_{50}, EC_{25}, and EC_{10} and 95% confidence interval (95% CI) are calculated for all six parameters**.**

**Parameters**	**EC_{50} (95% CI)**	**EC_{25} (95% CI)**	**EC_{10} (95% CI)**
Length	7.99 µM (7.4-8.75)	4.8 µM (4.43-5.18)	2.88 µM (2.49-3.28)
Area	5.2 µM (4.71-5.79)	3.2 µM (2.75-3.66)	1.97 µM (1.52-2.45)
Volume	4.51 µM (4.03-5.08)	2.91 µM (2.43-3.41)	1.88 µM (1.4-2.41)
Early-stage embryos	3.87 µM (3.51-4.24)	28 µM (2.76-3.71[a])	2.7 µM (2.06-3.35[a])
Late-stage embryos	1.51 µM (1.26-1.80)	1.26 µM (0.93-1.58[a])	1.05 µM (0.65-1.45[a])
Total embryos	3.08 µM (2.72-3.47)	2.27 µM (1.87-2.68)	1.67 µM (1.23-2.13)
[a]The 95% CI is calculated using symmetric asymptote.			

Supplementary Table 6. Strictly Standardized Mean Difference values for different concentrations of methylmercury. Strictly Standardized Mean Difference (SSMD) values were calculated by comparing the mean control wells for each treatment across 3 independent experiments for every methylmercury (MeHg) concentration. Green shading indicates excellent assay quality for a strong effect (SSMD ≥ 2).

MeHg (µM)	Length	Area	Volume	Early	Late	Total
10						
8	10.70	7.29	5.52	11.38	5.84	9.77
6	9.16	6.29	4.87	9.00	5.84	8.82
5	10.92	5.41	4.08	7.12	5.84	7.65
4	5.18	3.24	2.77	2.96	5.84	4.73
3.2714	3.58	2.40	2.12	1.05	5.74	3.04
2.6755	0.53	1.41	1.35	0.10	5.66	1.61
2.1882	-0.29	0.99	1.00	-0.32	3.53	1.00
1.7896	-0.91	0.34	0.41	-2.05	2.30	0.30
1.4636	-1.18	0.09	0.08	-2.37	1.03	-0.43
1.197	-2.05	-0.09	-0.10	-1.04	0.31	-0.30
0.979	-2.53	-0.24	-0.18	-0.64	-0.29	-0.49
0.8007	-3.42	-0.33	-0.22	-0.31	-0.33	-0.45
0.6548	-1.03	-0.38	-0.28	-0.67	-0.17	-0.57
0.5356	-1.89	-0.33	-0.34	-0.40	-0.68	-0.66
0.438	-1.23	-0.07	-0.02	-0.02	-0.62	-0.37
0.0876	-0.88	0.06	0.12	-1.09	-0.55	-0.89
0.0175	-0.57	0.11	0.19	-0.31	0.14	-0.11
0.0035	-0.79	0.00	0.12	-0.72	0.84	-0.08
0.0007	-0.86	0.07	0.19	-0.46	0.05	-0.21

Supplementary Table 7. Effective concentration values for DART-related parameters for propiconazole-treated worms. The EC_{10}, EC_{25}, and EC_{50} and 95% confidence interval (95% CI) are calculated for all six parameters. The EC_{10} values for data after normalization with respect to the controls are also shown.

**Parameters**	**EC_{50} (95% CI)**	**EC_{25} (95% CI)**	**EC_{10} (95% CI)**	**Normalized data EC_{10} (95% CI)**
Length	504 µM (392-685)	345 µM (320-388)	237 µM (192-294)	273 µM (237-308[a])
Area	384 µM (359-418)	223 µM (207-240)	130 µM (111-149)	108 µM (88-129)
Volume	336 µM (311-369)	188 µM (169-207)	105 µM (86-125)	93 µM (71-119)
Early-stage embryos	252 µM (230-276)	154 µM (132-175)	94 µM (72-117)	103 µM (82-125)
Late-stage embryos	78 µM (35-154)	33 µM (6-93)	14 µM (1-65)	12 µM (6-21)
Total embryos	204 µM (177-237)	109 µM (83-137)	58 µM (36-85)	57 µM (43-73)

[a]The 95% CI is calculated using symmetric asymptote.

Supplementary Table 8: Predefined assay acceptance criteria for DART experiments.

Parameter	Acceptance Criterion	Notes
Number of P_0 worms per experiment	4 L4 worms per plate, 2 plates per experiment	Transfer manually using standard picking technique
Removing of P_0 adults	All 4 P_0 adults removed from each plate at 48 hours	Terminate experiment if any P0 adult is missing
Bleaching efficiency	≥ 5,000 embryos obtained<br>≥ 50% hatching efficiency	Use synchronized L1s within 24 hours of bleaching
Number of L1s per well	80-100 L1s per well	Count prior to plating
Food concentration in the *C. elegans* liquid culture	OD_{600} = 3.0 ± 0.1	Measure the HB101 stock resuspended in S media using spectrophotometer
Incubator temperature	20 ± 0.1 °C	Monitor hourly via temperature sensor
Incubator humidity	40 – 60%	Monitor hourly via humidity sensor
C. elegans development in the solvent control	Worms in the vehicle control reach D1 adulthood	Manually verify worm number and developmental stages using a 2× objective
vivoChip imaging window	Capture vivoChip images 72 ± 1 hours after plating	Maintain consistent imaging time to reduce variability in DART parameters
Reference chemicals	Purchased from qualified vendor with documented batch number and Certificate of Analysis (CoA)	Follow vendor-specified storage and safety instructions
Master stock preparation	Prepare high concentration master stocks and store them at -80 °C for long-term storage and use	Prepare single use aliquots using DMSO or appropriate solvents
Working stock preparation	Prepare 200× fold dilutions for treatment	Store and use working stocks up to 3 days at 4 °C

References:

1. DuPlissis, A., et al., *Machine learning-based analysis of microfluidic device immobilized C. elegans for automated developmental toxicity testing.* Sci Rep, 2025. **15**(1): p. 15.
